# Computing the impact of central clearing on systemic risk

**DOI:** 10.3389/frai.2024.1138611

**Published:** 2024-02-21

**Authors:** Nikolai Nowaczyk, Sharyn O'Halloran

**Affiliations:** ^1^Independent Researcher, London, United Kingdom; ^2^Columbia SIPA and Political Science, Columbia University, New York, NY, United States; ^3^Trinity Political Science and Economics, Trinity College Dublin, Dublin, Ireland

**Keywords:** systemic risk, financial regulation, central clearing, artificial intelligence, credit risk, simulation, graph model, algorithm, G01, G18, G28

## Abstract

The paper uses a graph model to examine the effects of financial market regulations on systemic risk. Focusing on central clearing, we model the financial system as a multigraph of trade and risk relations among banks. We then study the impact of central clearing by *a priori* estimates in the model, stylized case studies, and a simulation case study. These case studies identify the drivers of regulatory policies on risk reduction at the firm and systemic levels. The analysis shows that the effect of central clearing on systemic risk is ambiguous, with potential positive and negative outcomes, depending on the credit quality of the clearing house, netting benefits and losses, and concentration risks. These computational findings align with empirical studies, yet do not require intensive collection of proprietary data. In addition, our approach enables us to disentangle various competing effects. The approach thus provides policymakers and market practitioners with tools to study the impact of a regulation at each level, enabling decision-makers to anticipate and evaluate the potential impact of regulatory interventions in various scenarios before their implementation.

## 1 Introduction

In the aftermath of the financial crisis, governments introduced a vast array of financial market regulations to mitigate the excessive risk-taking that, by many accounts, led to the collapse of the economic system. Central clearing has had mixed results, reducing default risk in some cases while increasing the incentive of market participants to take on more risks in others. Despite these regulations' uncertain effects, Former Federal Reserve Chair Janet Yellen asserted that “a lack of complete certainty about potential outcomes is not a justification for inaction,” see Yellen (2013).^1^

This raises the question on how to evaluate the impact of a financial regulation even before implementation. Relying on expert judgment alone to estimate the effect of regulations on financial markets is problematic as a consensus rarely arises, and reliable economic data is only available years after implementation. When discussing the potential impact of regulations, policymakers usually lack the tools and the data to assess policy alternatives, as when the G20 agreed to central clearing during the 2009 Pittsburgh summit, see G20 (2009). Today many excellent empirical studies, see e.g., Duffie and Zhu (2011) and Ghamami and Glasserman (2017), provide invaluable reviews of the impact of regulations on financial stability and risk reduction. These studies, however, depend on observational data, invariably confounding many correlated effects. Accordingly, isolating the impact of regulation by empirical analysis alone can be difficult and imprecise.

In order to fill this gap, we employ a graph model to predict and calculate cost (loss) functions, enabling us to evaluate the impact of different regulations on risk mitigation at both the firm and system levels. By mathematical analysis of that model, stylized and simulation case studies, we go beyond traditional expert judgment and empirical analyses, offering decision-makers a powerful tool to anticipate the economic effects of regulatory policies before implementation.

We apply our graph model approach to central counterparty clearing (CCP). Our analysis reveals that while central clearing can double the total levels of risk exposure in the cleared financial system, it also brings efficiency gains by automatically unwinding complex “daisy chains” of hedges. Moreover, our analysis uncovers the intricate interplay of netting benefits, gains and losses, the credit quality of clearing houses, and concentration risks, highlighting the multifaceted nature of risk dynamics at all levels of aggregation. By quantifying these offsetting forces individually, we can accurately predict the costs and benefits of various regulatory interventions in various case studies.

The remainder of the paper is organized as follows: we review the relevant literature in Section 2 placing our approach outlined in Section 3 into context. In Sections 3, 4, and 5 we present a theoretical study of the graph model illustrating the drivers of impact of the clearing regulation. We then apply this framework to stylized case studies in Section 6 and finally to a simulation case study in Sections 7 and 8. Finally, we summarize our findings, offer conclusions, and propose future research directions in Section 9.

## 2 Central clearing and systemic risk regulation

### 2.1 State of the art and review of literature

Despite its limitations, expert judgment-based research still remains useful as it can identify a wide range of possible effects. A good overview can be found in the aforementioned speech by the Former Federal Reserve Chair, Janet Yellen, see Yellen (2013). Of course, expert judgment cannot only be used beforehand, but also after a regulation has been phased in. For the impact of central clearing on systemic risk in particular, such qualitative approaches can be found in Kenyon and Green (2013).

Of course there are much more approaches to systemic risk through—in fact the literature on systemic risk in general is vast. A summary can be found in e.g., Silva et al. (2017) and an excellent comprehensive survey of systemic risk metrics can be found in Bisias et al. (2011). A key theme of both, the literature and the metrics, is default contagion, the risk that the default of one bank spreads to another. The study of how defaults spread is closely related to correlations of risk in the system and has been studied extensively, see e.g., Caccioli et al. (2015), Detering et al. (2016), and Pastorino and Uberti (2023).

The role of Central Counterparties (CCPs) in mitigating systemic risk in financial markets, particularly through the central clearing of over-the-counter (OTC) derivatives like credit default swaps, has become increasingly significant. CCPs act as intermediaries to manage default risk, using collateral and margin requirements to enhance financial stability. This process aims to diminish interconnectedness among market participants and bolster transparency. However, it also introduces potential risks, such as market concentration and increased transactional complexity. Key to this discourse is the impacts of CCPs on netting efficiency and collateral management. For a comprehensive review of this discourse see Berndsen (2021).

One of the most influential approaches, see e.g., Duffie and Zhu (2011) and Ghamami and Glasserman (2017), is to quantify ex-post the impact of the regulation after it has been implemented. This requires the collection of empirical risk data, which is then organized in a risk graph model, and some high-level distributional assumptions. These studies find that the intended positive impacts of central clearing are hampered by the fact that in practice, derivatives are not cleared through one central counterparty, but in fact through many different clearing houses. Obviously, empirical approaches yield interesting insights into the current state of the financial system, but cannot be used to evaluate future regulations. Another drawback of that approach is that empirical data is confounded by multiple effects. Even the clearing regulation itself does not only have the effect of introducing a central counterparty, but also introducing the requirement to post collateral, to contribute to the CCP default fund etc. And of course, the empirical data about the financial system collected after the financial crisis is also impacted by many other changes than just the introduction of clearing regulation, for example, liquidity buffer regulations.

### 2.2 Our approach: risk graphs computed from trade relation graphs

In theory, one could compute the impact of a regulation such as central clearing on the financial system, if one could obtain all the trade data of all the banks in a consistent format and then simulate the impact in one risk engine. In practice, this is, of course, not feasible as the trade data is proprietary, each bank stores this data in a different format and processes it through a different engine.

Nonetheless, it is possible to mathematically describe how a risk graph arises from a graph of trade relations between banks. The use of such an extended graph model facilitates the analysis of case studies, providing researchers with the ability to control trade relation data. This approach enables us to study the impact of a regulation like central clearing from the micro-economic bank level to the macro-economic aggregates in the financial system as a whole. And while even the most complex case study might not capture all details of the real financial system, this approach has various advantages: (i) it identifies the key drivers impacting a regulation; (ii) it enables analysis of various case studies; and (iii) it provides a tool to study the impact of the regulation before it is implemented providing policy makers with a meaningful insights.^2^

## 3 A graph model of systemic risk

This section introduces a graph model to describe trade relations in a financial system and the risks these trades induce. The basic idea is first to formalize the financial system FS as an undirected graph (*B, L*), in which the nodes *B* represent all the banks, and the links *L* represent all the trade relations. To each link ℓ ∈ *L* between any two banks *b*_1_ and *b*_2_, we then attach their trade portfolio τ(ℓ) as additional data, see **Figure 2A** for an example. In a second step, we associate to this *trade relation graph* FS a *risk graph* RG, which is a weighted directed graph (*B, A, w*). It contains the same banks as nodes *B*, but each trade relation ℓ between any two banks *b*_1_ and *b*_2_ is replaced by two arrows *a*_12_, *a*_21_ ∈ *A* pointing in opposite directions. These arrows represent the risk induced from *b*_1_ onto *b*_2_ and vice versa due to their trades; see **Figure 2B** for an example. The weights *w*_12_ and *w*_21_ on these arrows quantify the risks in a chosen risk metric. In the third step, we aggregate the risks from the links to the nodes and the nodes to the entire system by computing weighted degrees—a pure graph theoretical method. We summarize the three steps in the following subsections. We assume the reader to be familiar with basic notions of graph theory; see the Supplementary Appendix A.1 for essential definitions and notation. A purely mathematical formulation of the graph model can also be found in Supplementary Appendix A.2.

This graph model has three key features, making it particularly suited for our analysis: first, notice that the risk relations are not sourced from empirical data and given as an input to the model; the risk graph is an output from the model. Second, the input to the model is the underlying graph of trade relations, which is not stylized or simplified but fully specified in detail using an XML format, which a simulation engine can transparently process. Third, the formalism by which we describe the input trade relations mathematically is an abstraction of those concrete XML representations, which describe the input technically. Thus, this approach has the advantage that the mathematical theory and the technical implementation are closely aligned.

### 3.1 Financial systems as trade relations graphs

We model a financial system FS as an undirected multigraph of trade relations,^3^ see **Figure 2A** for an example. More precisely, a *financial system* is a tuple FS = (*B, L*, τ, β), where the set *B* represents all the banks in the system. For every trade relation between any two banks *b*_1_, *b*_2_ ∈ *B*, there is precisely one link ℓ = {*b*_1_, *b*_2_} ∈ *L*. The collection *L* of all these trade relations is a multiset, i.e., we allow banks to have multiple parallel trade relations; thus (*B, L*) is an undirected multigraph. We used undirected graphs for this model because the property of being in a trade relation is symmetric. We then assign to each trade relation link ℓ a netting set τ(ℓ) = {*y*_1_, …, *y*_*k*_} of trades, where each trade is an element in some trade data space *Y*. Thus, mathematically τ is a function τ:*L* → [*Y*], ℓ↦τ(ℓ), where *Y* is a space representing all the possible trades in the financial system and [*Y*] denotes the set of finite subsets of *Y*. This space is discussed in more detail in Section 3.2 below. Optionally, we can also define a node data function β:*B*→*X*, which assigns to each bank *b* ∈ *B* additional data β(*b*) (for instance, core capital) in some node data space *X* (no node data function exists in **Figure 2A**).

From a risk perspective, having more than one trade relationship is never beneficial for either counterparty. Still, valid economic and operational reasons exist for two banks to have more than one netting set of trades.

### 3.2 The trade relation space

Modeling the trade data space *Y* in a precise yet tractable fashion is a difficult task every bank in the derivatives business faces. On the one hand, the space *Y* needs to be rich enough to accurately represent every OTC derivative the bank might want to trade. On the other hand, it still needs to be technically tractable to process all the derivatives traded through the bank's IT systems. In practice, this problem is usually solved using an internal proprietary standard, which is not available for academic research.

One possible alternative is the use of open source formats, for example ORE XML, which is compatible with an open source risk engine, see ORE XML and ORE. Thus, for this article, particularly the simulations described in Section 7, the space *Y* is the space of all valid trade representations in ORE XML format. An example of such a representation is shown in **Figure 8**.

Our study of central clearing rests on theoretical considerations and numerical simulation. In numerical simulations, the XML representation of the trades can be processed directly. For theoretical considerations, adding the entire content of an ORE XML file to a graphical representation such as **Figure 2A** or mathematically formalizing this XML format in all details is theoretically perfectly possible but quite cumbersome. Also, most trade details are not needed for most of the mathematical derivations in central clearing. For the analysis in Section 4, we only need a formalism to describe which counterparty has which side of the deal.

Notation convention: to that end, we assume that all trades in a financial system FS are enumerated, and we denote them by *T*_1_, *T*_2_, …. We consider two trades, *T* and *T*′, equivalent if their only difference is the counterparty entry, i.e., which bank in the system is long and which is short. We also assume that the equivalence classes of trades, the *trade templates*, are enumerated as well and are denoted by $T_{1},T_{2},\ldots$. For any two banks *b*_1_, *b*_2_ in the system, we then denote by $T_{1}\langle b_{1},b_{2}\rangle$ the trade, in which *b*_1_ is inserted into $T_{1}$ as the first counterparty and *b*_2_ as the second. Notice that this template notation extends canonically to a netting set by defining


(1) $$\begin{array}{l} \left\{T_{1},T_{2},\ldots \right\}\langle b_{1},b_{2}\rangle :=\left\{T_{1}\langle b_{1},b_{2}\rangle ,T_{2}\langle b_{1},b_{2}\rangle ,\ldots \right\}. \end{array}$$

An example of this notation is shown in **Figure 2A**. This notation allows us to illustrate neatly which counterparty is on which side of a deal but does not require any formalism around any of the other trade details.^4^ Another critical advantage of this convention is that it creates a strong link between mathematical theory and practical implementation.

### 3.3 Risk metrics

We have modeled a financial system FS as a trade relation graph FS = (*B, L*, τ, β). Next, we want to model the various risks in that system. To that end, we need to specify appropriate risk metrics. Even if we only consider two banks *b*_1_, *b*_2_ ∈ *B* in a trade relation ℓ = {*b*_1_, *b*_2_} ∈ *L* with netting set *s* = τ(ℓ), the problem of assigning a single number *w*(*s*) ∈ ℝ_≥0_ to this netting set that accurately reflects its risks is a non-trivial task. How to choose *w*?

First, one should remark that there are different types of risk, for example, market, credit, liquidity, operational, etc. For each type of risk, practitioners use many micro-prudential risk metrics. Because regulators need to treat banks fairly, metrics, which need to be reported to the regulator, are standardized. In this article, we will focus on counterparty credit risk, and we will use two regulatory standard metrics: (i) EEPE, the *effectivized expected positive exposure* and (ii) CVA, the *unilateral credit value adjustment*. The counterparty credit risk to bank *b*_1_ in a trade relation ℓ = {*b*_1_, *b*_2_} is the risk that *b*_2_ defaults before it has met all its obligations resulting from the derivative trades τ(ℓ) to *b*_1_. The metric EEPE is a measure of exposure; it roughly states the dollar amount lost over the next year if the counterparty defaults but does not consider the default probability. The CVA integrates the exposure against the probability of default PD of the counterparty and can thus be considered an expected loss. The precise implementation of EEPE and CVA used in the simulation can be found in the ORE user guide,^5^ see ORE, Supplementary Appendix A.3.

The range of metrics in that model can easily be extended to capture more exposure metrics. In fact, under mild technical conditions, one can think of any function which assigns a number to each netting set as a risk metric; see Supplementary Appendix A.3 for a precise technical formulation. A fundamental property of exposure, though, is that it only depends on the trades in the netting set and not the counterparties, where CVA also depends on the counterparty.

### 3.4 Risk graph

Given a financial system FS = (*B, L*, τ, β) and a risk metric *w*, we can organize all the risks of all the trades in the system in a new graph, called the *risk graph* RG = RG(FS).

More precisely, the *risk graph* of FS for the metric *w* is a tuple RG = (*B, A, w*), where the nodes *B* are again the banks. For each link ℓ = {*b*_1_, *b*_2_} ∈ *L* in the trade relation graph, there are two arrows *a*_12_ = (*b*_1_, *b*_2_), *a*_21_ = (*b*_2_, *b*_1_) ∈ *A* pointing in opposite directions in the risk graph. These arrows represent the risk the banks are inducing on each other due to their trades. Finally, on each arrow *a*_12_ we attach the risk *w*_12_ in τ({*b*_1_, *b*_2_}) induced from *b*_1_ onto *b*_2_ measured in metric *w* and vice versa. By slight abuse of notation, we now also think of *w* as a function *w*:*A* → ℝ_≥0_.

For example, the risk graph resulting from the trade relation graph in **Figure 2A** is shown in **Figure 2B**. The key difference in shape is that each link is replaced by two arrows pointing in opposite directions. In case two banks *b*_*i*_ and *b*_*j*_ have multiple trade relations, we denote by $w_{ij}^{\left(k\right)}$ the weight attached to the arrow (_*b*_*i*_, *b*_*j*_)*k*_ ∈ *A* resulting from the trades attached to the *k*-th link {_*b*_*i*_, *b*_*j*_}*k*_ ∈ *L*.

Notice that weights $w_{ij}^{\left(k\right)}$ and $w_{ji}^{\left(k\right)}$ attached to two opposing arrows (_*b*_*i*_, *b*_*k*_)*j*_, (_*b*_*j*_, *b*_*i*_)*k*_ ∈ *A* might be very different even though they result from the same trades τ({_*b*_*i*_, *b*_*j*_}*k*_). That is because the two sides of the deals can induce very different amounts of risk measured in the risk metric *w*: an interest rate swap, for example, is a two-sided derivative, which induces (different amounts of) credit risk on both sides. In a one-sided trade like an option, the issuer of the option is not exposed to any credit risk. In contrast, the buyer of the option is fully exposed to potentially significant credit risk.

### 3.5 Aggregation

The risk graph RG = (*B, A, w*) contains all risk data measured in metric *w* attached to the links. This micro-prudential information has the advantage that it is very rich. In the next step, we want to aggregate this information to a macro-prudential level. To that end, we apply a pure graph theoretic method, called the *weighted degree*, to aggregate the risk metrics from the arrows to the nodes and then from the nodes to the system.

For any node *b* ∈ *B*, we define


(2) $$\begin{array}{l} w^{\pm }\left(b\right):=\underset{a\in A^{\pm }\left(b\right)}{\sum }w\left(a\right), \end{array}$$

where *A*^±^(*b*) are all the arrows that start/end at *b*. Thus, *w*^±^(*b*) is the total risk induced/received by the bank *b*. We define


(3) $$\begin{array}{l} w_{\mathrm{tot}}:=w\left(\mathrm{RG}\right):=\underset{b\in B}{\sum }w^{\pm }\left(b\right)=\underset{a\in A}{\sum }w\left(a\right) \end{array}$$

as the total amount of risk in the system. This in itself constitutes a metric of systemic risk and also allows us to define the relative node metrics


(4) $$\begin{array}{l} \rho^{\pm }\left(b\right):=\frac{w^{\pm }\left(b\right)}{w_{\mathrm{tot}}}. \end{array}$$

These express the risk induced/received by *b* as a fraction of the total and are thus suitable to detect the concentration of risk.

### 3.6 Correlation

While using *w*_tot_ as a metric of systemic risk, i.e., defining systemic risk as the sum of all risks in the system, is a straightforward aggregation, it is certainly not without drawbacks as this metric ignores the correlation structure in the risks. Even for a single bank, the aggregation of the risks from the links to the nodes by summation ignores the correlation structure of the various netting sets of trades on its links. This implicitly assumes that all risks correlate +1, i.e., no risks ever offset each other.^6^ We are choosing this convention because this assumption is conservative and a standard business practice in risk management accepted by regulators.

Mathematically, it is perfectly possible to estimate a correlation matrix between the risks stemming from the netting sets on the links and then aggregate the results to the nodes by weighing the risk weights with that correlation matrix. Analogously, one can aggregate the risks from the nodes to the system. However, obtaining a reasonable estimate for those correlations is a non-trivial matter. The study of correlations of risks is nevertheless an active and exciting field of research in its own right and particularly relevant for the study of default contagion, see for example Cont et al. (2013), Detering et al. (2016), and the overview in Bisias et al. (2011).

## 4 Clearing and trade relations

This section will formalize central clearing using the graph model developed in Section 3. Starting with a trade relation graph of a given bilateral financial system FS = (*B, L*, τ, β), we first define clearing as an operator on that graph. As clearing is quite a complex operation, we describe this *clearing operator* in three steps called *repartitioning, pre-clearing*, and *compression*. All steps are visualized in an example, see **Figure 2**, and explained in the following subsections. A purely mathematical formalization is given in Supplementary Appendix A.4.

**Figure 1:**
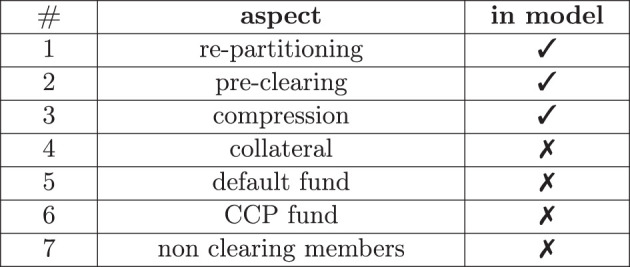
Aspects of clearing.

Clearing houses have more aspects than simply centralizing trades; see Figure 1 for an overview. Modeling all of these aspects simultaneously is not only formally cumbersome, but it is also not insightful since, ideally, we would also like to understand the effect of every aspect separately. Sticking to the first three has the advantage that this is the minimum scope required to gain insight into the key ideas of clearing, prominently discussed in Yellen (2013), without diluting the result by aspects that conceptually have nothing to do with clearing. For example, collateralization can also be implemented in a bilateral system without clearing the trades first; see O'Halloran and Nowaczyk (2019) for our earlier studies on that. In our model, neither the bilateral nor the cleared system is collateralized, as we aim to understand the effect of clearing in isolation from all other effects.

**Figure 2:**
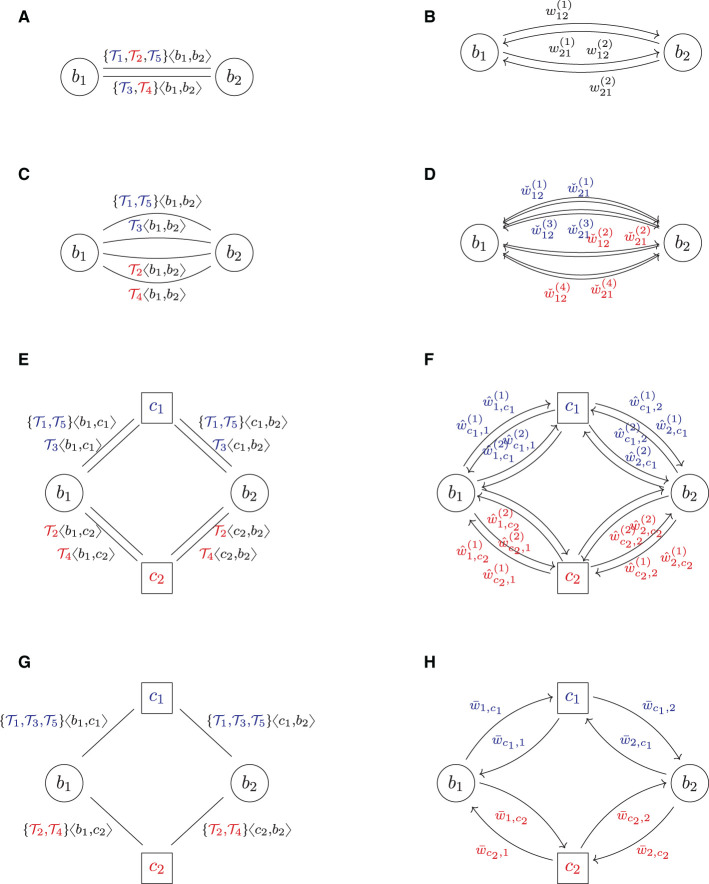
Multi clearing (colors indicate asset class). **(A)** FS: trade relation graph (bilateral). **(B)** RG: risk graph (bilateral). **(C)** $\overset{ˇ}{\text{FS}}$^(2)^: trade relation graph (repartitioned). **(D)** $\overset{ˇ}{\text{RG}}$^(2)^: risk graph (repartitioned). **(E)** ${\hat{\mathrm{FS}}}^{\left(2\right)}$: trade relation graph (multi pre-cleared). **(F)** ${\hat{\mathrm{RG}}}^{\left(2\right)}$: risk graph (multi pre-cleared). **(G)** ${\bar{\mathrm{FS}}}^{\left(2\right)}$: trade relation graph (multi cleared). **(H)** ${\bar{\mathrm{RG}}}^{\left(2\right)}$: risk graph (multi cleared).

The key idea of central clearing is as follows: let FS = (*B*, L, τ, β) be a financial system; see, for example, Figure 2A. Assume that a bank *b*_1_ has a bilateral derivative contract $T\langle b_{1},b_{2}\rangle$ with a bank *b*_2_. Then *clearing* this trade through a clearing house *c*, often called *Central Counterparty (CCP)*, means that the derivative is broken up into two contracts: one contract $T\langle b_{1},c\rangle$ between *b*_1_ and *c* and another contract $T\langle c,b_{2}\rangle$ between *c* and *b*_2_. From the perspective of both *b*_1_ and *b*_2_, the new contract has the same terms and conditions and cash flows. The only difference is that they no longer face each other but the CCP. In the bilateral setting, if *b*_2_ defaults, then *b*_1_ suffers a loss. In the cleared setting, if *b*_2_ defaults, then *b*_1_ still has its contract with the CCP and suffers no direct loss. The CCP might suffer a loss from its deal with *b*_2_, though.

### 4.1 Repartitioning

The problem with that idea is that it cannot be put into practice: in reality, no truly central counterparty exists. After all, which country would host and regulate an institution that takes one side in all derivative trades worldwide? The clearing business is partitioned by geographic location, currency, and asset class. The empirical study Ghamami and Glasserman (2017) finds that five of the ten largest US banks use 17 CCPs. Thus, in practice, *central clearing* is *multi clearing*.

To make things worse, one must consider that the bilateral trade relations between banks usually involve *netting agreements*, i.e., the agreement to net outstanding debit and credit of a whole portfolio of derivatives in case of a default. The structure of these netting agreements is generally incompatible with the structure of the clearing house market. For instance, two banks might have a netting set with multiple asset classes, e.g., interest rate derivatives and credit derivatives, and thus, may net their bilateral exposures stemming from all of these trades. As clearinghouses typically only clear one asset class, it cannot be cleared directly.

The solution to this problem is that first, the two banks have to *repartition* their netting sets per asset class. This can be formalized as follows: let Γ = {γ_1_, …, γ_*m*_} be a set of asset classes and α:*Y* → Γ be a function that assigns each trade *y* its asset class γ(*y*). In the example shown in Figure 2A, we assume that there are *m* = 2 types of asset classes, say IR/FX (interest rate and foreign exchange) and EQ (equities), which is shown in blue and red in the trade relation graph Figure 2A. Now, *repartitioning* a netting set with respect to the asset classes assigned by α means to break up the existing netting set of trades and instead form a new netting set for each asset class γ ∈ Γ. We formalize this as an operator RP_α_:FS ↦ $\overset{ˇ}{\text{FS}}$, which applies this repartitioning to every netting set in the system and thus transforms the initial system FS into a repartitioned system $\overset{ˇ}{\text{FS}}$. As exemplified in Figure 2C, the result is a financial system in which every netting set comprises of precisely one asset class. Notice that this process creates many parallel links in the system.

### 4.2 Preclearing

In a second step, which we call *pre-clearing*, the banks can now break up each such netting set and clear it through precisely one CCP. In our model, we assume that there is a fixed set of *m* asset classes Γ = {γ_1_, …, γ_*m*_} and that for each asset class γ_μ_, there is precisely one CCP *c*_μ_, which clears all trades of asset class γ_μ_. Applying this to every netting set in the system defines an operator $\hat{\mathrm{MC}}:\overset{ˇ}{\text{FS}}\mapsto \hat{\text{FS}}$. An example of the result is shown in Figure 2E: as there are *m* = 2 asset classes, we can see *m* = 2 CCPs.

### 4.3 Compression

The system resulting from the pre-clearing is usually a system with many parallel links, i.e., the banks have multiple netting sets with the same clearing house resulting from its (formally bilateral) business with multiple counterparties. As this is unfavorable to both the bank and the clearing house, these netting sets get *compressed* into one netting set in a third step. The legal process of breaking up a netting set of deals (potentially of mixed asset classes) and clearing them through (potentially multiple) central counterparties, and then compressing them into one netting set is also called *novation* and is a non-trivial matter.

Formally, the compression can also be seen as an operator $\text{CMP}:\overset{ˇ}{\text{FS}}\mapsto \bar{\text{FS}}$, which removes all parallel links between any two counterparties and compresses all the trades attached to these parallel links into one big netting set. The compression of the example in Figure 2E is shown in Figure 2G. The resulting system then has no parallel links anymore.^7^

Finally, we define *clearing* as the composition


$$\bar{MC}:\;=\text{ CMP}\circ \hat{MC}\circ {\text{ RP}}_{\alpha }:\text{ FS}\mapsto \bar{\text{FS}}$$

of these three operators.

Notice that while in the multi-cleared system in Figure 2G, each bank still has two netting sets with multiple trades, they are now netted in the other combination as in the bilateral system. In the cleared system, realized netting benefits across asset classes within the same netting set are now lost, but netting benefits within the same asset class across different counterparties are gained.

While the number *m* will always be much bigger than one in practice, the case *m* = 1 is still mathematically and conceptually insightful as it reflects precisely the original idea of clearing as considered in, e.g., Yellen (2013). This case has a different flavor as repartitioning is not necessary. This case is visualized in the Supplementary Figure 18. The centrally cleared system Supplementary Figure 18e has the property that each bank *b* still has the same trades as before. Still, instead of having them in multiple netting sets with multiple counterparties, it now has them compressed into a single netting set with the CCP. This yields a drastically different netting structure in the cleared system, which benefits the bank.

### 4.4 Other aspects

We summarize the remaining aspects of clearing from Figure 1 and the reasoning behind them even though they are not part of the model: after clearing in the above sense, the member banks of a clearing house are now no longer exposed to any other member, but the CCP is exposed to the default to all of them. To mitigate this risk, the CCP will call its members for collateral in the form of variation and initial margin. Notice that *collateralization* is a second regulation that has also been introduced since the 07/08 crisis for both cleared and uncleared derivatives.

In case of a clearing member default, the CCP can liquidate that member's collateral. However, even if a clearing member is well over-collateralized at default, the collateral might still not be sufficient to cover the losses. To that end, all clearing house members must pay into a *default fund*, which can be used to absorb those losses. The CCP must also pay into a so-called *skin-in-the-game fund*, which absorbs further losses. The purpose of that fund is to incentivize the CCP to call for enough collateral to avoid suffering its losses. All remaining clearing members can save the distressed CCP with their remaining funds if this is still insufficient. The CCPs and the banks set the precise terms and conditions of this default waterfall which vary between CCPs.

Finally, it should be highlighted that only the big banks are usually direct members of the clearing house. If two smaller banks enter a derivative deal, they can each nominate a big clearing member bank to clear the derivative on their behalf, complicating the clearing process further.

## 5 Clearing and risk graphs

Starting from a bilateral system of trade relations, we have described how to construct its clearing as the composition of three operators: repartitioning, pre-clearing, and compression. All these operators change the trade relation graph of the financial system. We now want to study the impact of these changes on the risk graph associated with the trade relation graph. We first stick to counterparty credit risk exposures (EEPE) and then discuss CVA. These effects are visualized in the right column of Figure 2.

**Figure 3:**
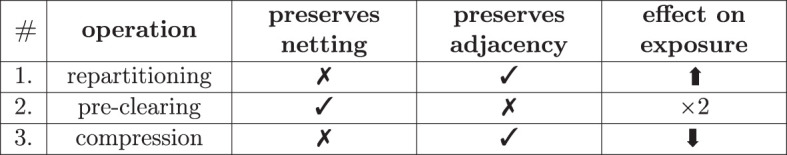
Effect of operators.

Before we derive the quantitative *a priori* estimates of the clearing operators in this section, we describe the impact qualitatively; see the summary in Figure 3: the repartitioning always increases exposures. This is because breaking up an existing netting set always results in a loss of netting benefits. At worst, this loss can be arbitrarily large, and at best, it is zero, but it will always be a loss. This crude *a priori* estimate cannot be improved upon without detailed knowledge of the trade data of the netting set. The only exception is *m* = 1, i.e., if everything is cleared through one central counterparty. In this case, this operator has no effect and can be ignored.

The impact of pre-clearing, however, can be given precisely *a priori*: the pre-clearing operator splits every netting set into two and inserts a CCP in the middle. Thus, after pre-clearing, every bank in the system has the same risk exposures as before but is now facing the CCP. As the new players, The CCPs face the banks and, in total, have the same exposures as them. Therefore, the total amount of exposure in the system has doubled. This can also be seen graphically by comparing Figure 2E with Figure 2D: we can see that the number of arrows has doubled, and one can show that every risk weight now occurs twice.

After pre-clearing, the banks typically have many netting sets with the clearing house. The compression operator compresses all of these into a single netting set. This yields netting benefit gains and thus reduces the risk in the system again. The precise amount of reduction also cannot be given *a priori* as it depends on the trade and netting details.

### 5.1 Total levels of exposure

Formally, these insights can be summarized in the following Theorem.

**Theorem 1 (effect of clearing on total risk levels)**. Let FS = (*B*, L, τ) be a bilateral trade relation graph and assume that FS has already been repartitioned. Then the total levels of exposure^8^ $w_{\mathrm{tot}}^{\mathrm{bil}}$, $w_{\mathrm{tot}}^{\mathrm{pre}}$, $w_{\mathrm{tot}}^{\mathrm{cleared}}$ in the bilateral system, the pre-cleared system, and cleared system are related by


(5) $$\begin{array}{l} 0\leq w_{\text{tot}}^{\text{cleared}}\leq w_{\text{tot}}^{\text{pre}}=2w_{\text{tot}}^{\text{bil}}. \end{array}$$

Both estimates are sharp.

A Supplementary Appendix provides a slightly more general version of that theorem with more technical details and a formal proof; see Supplementary Theorem A.21.

The interpretation of Equation (5) is as follows: at worst, the compression operator does not unleash any netting benefits. In this case, the pre-clearing simply doubles all the exposures, and the cleared system has twice as much risk as the bilateral system. At best, the compression operator unleashes so many netting benefits that all risks in the system net each other out, and the cleared system has no risk anymore. In general, some risks will net out while others do not. We will present examples for all three cases in the case studies in Section 6, which proves that these estimates are sharp.

The result depends on the chosen metric *w* and its aggregation. It holds for exposure but not for CVA, as CVA also depends on the counterparty's credit quality. The result also depends on the aggregation logic, recall Equation (3), and thus on maximally conservative assumptions on the correlation of risks. If one chooses a systemic risk metric considering the correlation structure, the situation would be even more subtle.

Notice that for *m*>1, the results of Theorem 1 only hold if we assume that the system is already repartitioned. As for *m*>1, this is usually not the case. Whether or not a multi-cleared system is safer depends subtly on the ratio of the loss in cross-asset netting benefits resulting from the repartitioning vs. the cross-counterparty netting benefits gains from the compression. We study this effect in our numerical simulation, see Section 8.

### 5.2 Netting benefits and transparency

Of course, the relationship Equation (5) is not what one would intuitively hope for. What we would like to know is if


(6) $$\begin{array}{l} 0\leq w_{\text{tot}}^{\text{cleared}}<w_{\text{tot}}^{\text{bil}}, \end{array}$$

i.e., if the cleared system has less risk than the bilateral system (and not just less than twice the bilateral system).

We already know this question cannot be answered *a priori* because the bounds in Equation (5) are sharp. However, one can make precise the notion of netting benefits and work out a criterion to check this equation that is a bit more accessible.

To that end, we define for any bank *b* ∈ *B* its *netting benefit*


(7) $$\begin{array}{l} \Delta_{w}^{\pm }\left(b\right):=w^{\pm }\left(b\right)-{\bar{w}}^{\pm }\left(b\right), \end{array}$$

(8) $$\begin{array}{l} \;={ŵ}^{\pm }\left(b\right)-{\bar{w}}^{\pm }\left(b\right) \end{array}$$

i.e., the difference between the risk *w*^±^(*b*) induced/received by *b* in the bilateral system and the risk ${\bar{w}}^{\pm }\left(b\right)$ induced/received in cleared system. Because we are assuming that the system is repartitioned (in particular for *m* = 1), we have *w*^±^(*b*) = ŵ^±^(*b*), i.e., the risks ŵ^±^(*b*) for *b* in the pre-cleared system are the same as in the bilateral system, which justifies the second equation, Equation (8). This formulation has the advantage that it is meaningful for the banks and the CCPs.

Using this notion, we obtain:

**Theorem 2 (netting benefits)**. Under the same hypothesis as in Theorem 1, the cleared system has less risk than the bilateral system if and only if the netting benefits of all banks are bigger than the risk introduced by all CCPs, i.e., Equation (6) holds if and only if


(9) $$\begin{array}{l} \underset{b\in B}{\sum }\Delta_{w}^{\pm }\left(b\right)>\overset{m}{\underset{\mu =1}{\sum }}{\bar{w}}^{\pm }\left(c_{\mu }\right). \end{array}$$

Some further characterizations of this equivalence are in the Supplementary Theorem A.24.

Whether or not the cleared system has less exposure than the bilateral system depends subtly on the topology of the trade relation graph, the netting structure, the bilateral risk metric *w*, and its aggregation logic. Thus, this is a global property, and it is hard to decide whether or not it holds. The characterization Equation (9) is useful as it breaks this global property down into local aggregations. Computing the netting benefits of each bank allows us to understand at what points in the system we see a reduction in exposure and at which points we do not.

Who could verify Equation (9) in practice? While each bank *b* ∈ *B* can compute its netting benefits $\Delta_{w}^{\pm }\left(b\right)$, it cannot know the netting benefits of any other counterparties in the system. The key insight into netting benefits is that they are symmetric, i.e., the total netting benefits gained by all banks are equal to the total netting benefits gained by all the CCPs.

**Theorem 3 (netting benefit symmetries)**. Under the hypothesis of Theorem 1, it holds that


(10) $$\begin{array}{l} \underset{b\in B}{\sum }\Delta_{w}^{\pm }\left(b\right)=\overset{m}{\underset{\mu =1}{\sum }}\Delta_{w}^{\pm }\left(c_{\mu }\right). \end{array}$$

Technical proofs are given in the Supplementary Corollary A.26.

If we combine the result Equations (9), (10), we obtain the following:

**Corollary 4 (transparency)**. Under the hypothesis of Theorem 1, Equation (11) holds if and only if


(11) $$\begin{array}{l} \overset{m}{\underset{\mu =1}{\sum }}\Delta_{w}^{\pm }\left(c_{\mu }\right)>\overset{m}{\underset{\mu =1}{\sum }}{\bar{w}}^{\pm }\left(c_{\mu }\right). \end{array}$$

The Equation (11) is a good example of the transparency argument made by proponents of clearing: the information about the system required to answer a question, in this case, whether or not Equation (6) holds, is available in the CCPs already and does not need to be aggregated from individual bank data. However, this is also a good example of how this argument becomes weaker the more CCPs are used in the system. Only if all trades are cleared through one central clearing house, this CCP is indeed also a single point of knowledge.

### 5.3 Netting benefits and concentration risk

On the other hand, opponents of central clearing argue that a central clearinghouse creates a single point of failure for the financial system. Even when multiple clearing houses are used, the CCPs can become very big entities in the financial system.

How much exposure is concentrated in the CCPs after clearing? The exposure concentration in the pre-cleared system can be easily stated: by definition of the pre-clearing operator, all CCPs mirror the netting sets of the banks and thus, the percentage share of the risks ${ŵ}^{\pm }\left(c_{\mu }\right)$ of the various CCPs is given by


$$\begin{array}{l} {\hat{\rho }}^{\pm }\left(c_{1},\ldots ,c_{m}\right):=\frac{\overset{m}{\underset{\mu =1}{\sum }}{ŵ}^{\pm }\left(c_{\mu }\right)}{w_{\mathrm{tot}}^{\mathrm{pre}}}=\frac{1}{2}. \end{array}$$

The situation appears more subtle in the cleared system as the compression unleashes netting benefit gains for the banks and the CCPs. However, by Equation (10), these gains are symmetric. Thus, in the cleared system, this equation holds too:

**Corollary 5 (concentration risk)**. Let FS be a financial system. Then the exposures ${\bar{w}}^{\pm }\left(c_{\mu }\right)$ of all the CCPs constitute half of the risk in that system after clearing, i.e.,


$$\begin{array}{l} {\bar{\rho }}^{\pm }\left(c_{1},\ldots ,c_{m}\right):=\frac{\overset{m}{\underset{\mu =1}{\sum }}{\bar{w}}^{\pm }\left(c_{\mu }\right)}{w_{\mathrm{tot}}^{\mathrm{cleared}}}=\frac{1}{2}. \end{array}$$

The Supplementary Appendix discusses these technicalities; see Equation (49) in Supplementary Appendix.

The interpretation is that for *m* = 1, half of the exposure in the system is concentrated in the CCP, which confirms the argument that a clearing house results in a high concentration of exposures.

### 5.4 Effect on CVA

Notice that the hypothesis of Theorem 1 requires *w* to be a bilateral exposure metric like EPE(*t*) or EEPE. It does not hold for CVA as CVA is defined as an average trade exposure weighted by the probability of default (PD) of the counterparty and thus depends on the counterparty. Thus, this theorem is not directly applicable. Exposure metrics like EEPE only consider a loss-given default but no probability of default. But obviously, the probability of default is a key quantity in credit risk. Thus, measuring the impact of central clearing on a financial system in terms of CVA is very interesting. However, this poses a difficult methodological challenge: How to compute a clearing house's default probability?

The usual method, see for example Brigo and Mercurio (2006), Chan-Lau (2006), and Lichters et al. (2015), of stripping the PD of a counterparty from CDS quotes requires a liquid market of actively traded CDS for that counterparty. However, there are no credit default swaps on CCPs, and thus, no market to estimate its default probability, PD. While before the 2007/08 crisis, the AAA-rated bank was the entity in the financial system of which we assumed it could not default, in the cleared world one might be under the impression that the CCP is now assumed to be theoretically unable to default. If we take that seriously, then the PD of the CCP is zero. Consequently, the CVA of all banks in the system is also zero. The CCP would still be exposed to non-trivial CVA, but if it cannot default no matter what, why should one be bothered about its risks?

There are historical examples of defaulted clearing houses; thus, it cannot be assumed that CCP's default probability is zero. There is currently a vivid debate about how one should compute it, and regulators seem to take the view that a clearing house can default even though the probability of this event is very low, see for example, the discussions in Sherif (2016), Arnsdorf (2019), and Ryder (2019).

Since settling these questions is beyond the scope of this text, we restrict our scope to exploring the impact of the PD of the CCP on systemic CVA via a numerical simulation, see Section 8.

## 6 Stylized case studies

In this section, we discuss a few simple comprehensible examples of financial systems, which illustrate the formalism developed in Section 4 and give examples of systems where the bounds given in Equation (5) are sharp. Some additional examples of star-shaped, complete, and bipartite trade relation graphs and their mathematical properties are discussed in Supplementary Appendix A.5.

In all these examples, we will assume that *w* is a bilateral exposure metric. We denote the risk of a bank *b* in the bilateral, pre-cleared and cleared system by *w*^±^(*b*), ŵ^±^(*b*), respectively ${\bar{w}}^{\pm }\left(b\right)$.

While the examples in the case studies are too small to be realistic, they provide structural insight into the problem. This technique has been used in earlier work to study the impact of collateralization; see O'Halloran et al. (2017). We focus entirely on the case *m* = 1, i.e., on central clearing.

### 6.1 Two banks

**Figure 4:**
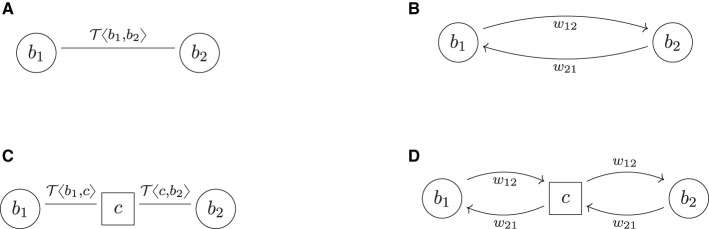
Two banks. **(A)** Trade relation graph (bilateral). **(B)** Risk graph (bilateral). **(C)** Trade relation graph (cleared) **(D)** Risk graph (cleared).

The simplest example of a financial system FS = (*B, L*, τ) is two banks in one trade relationship with a single trade, see Figures 4A, B. The application of the central clearing operator yields Figures 4C, D. We conclude that $w^{\pm }\left(b_{i}\right)={ŵ}^{\pm }\left(b_{i}\right)={\bar{w}}^{\pm }\left(b_{i}\right)$, *i* = 1, 2, i.e., both banks have the same risks in the bilateral, the pre-cleared and the cleared system. Central clearing does not realize any additional netting benefits for either of the two banks, but the CCP induces new risk. Therefore, $w_{\mathrm{tot}}^{\mathrm{cleared}}=2w_{\mathrm{tot}}^{\mathrm{bil}}$, i.e., this is an example where the upper bound in Equation (5) is sharp.

### 6.2 The perfect hedge

**Figure 5:**
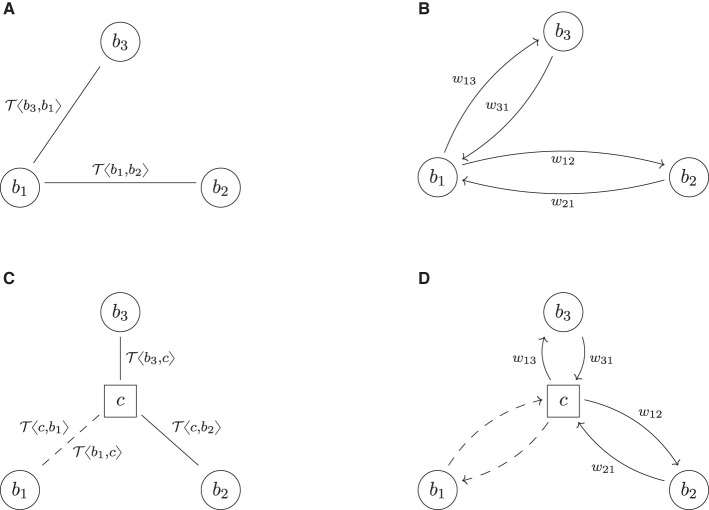
The perfect hedge. **(A)** Trade relation graph (bilateral). **(B)** Risk graph (bilateral). **(C)** Trade relation graph (cleared). **(D)** Risk graph (cleared).

We now add a third bank into the system and assume that *b*_1_, which has the trade $T\langle b_{1},b_{2}\rangle$ with *b*_2_, now enters into a perfect hedge^9^ $T\langle b_{3},b_{1}\rangle$ of that deal with *b*_3_. The bilateral trade relation and risk graphs and their central clearing are shown in Figure 5. In the cleared system, *b*_1_ can realize its netting benefits across the two counterparties, which is impossible in the bilateral system. Because the deals are perfect hedges of each other, the result is that they cancel out. The netting set $\left\{T\langle b_{1},b_{2}\rangle ,T\langle b_{3},b_{2}\rangle \right\}$ is equivalent to the empty netting set from the perspective of *b*_1_ (and by symmetry also for the CCP). Thus, $0={\bar{w}}^{\pm }\left(b_{1}\right)<{ŵ}^{\pm }\left(b_{1}\right)=w^{\pm }\left(b_{1}\right)$. For the other banks *b*_2_ and *b*_3_, no netting benefits can be realized and we obtain ${\bar{w}}^{\pm }\left(b_{i}\right)={ŵ}^{\pm }\left(b_{i}\right)=w^{\pm }\left(b_{i}\right)$, *i* = 2, 3.

Notice that in the cleared system, from which we can effectively remove *b*_1_, the remaining counterparties *b*_3_, *c*, and *b*_2_ are now in the same logical trade relationship as *b*_3_, *b*_2_ and *b*_1_ in the bilateral system, i.e., the risk graphs Figures 5B,D are isomorphic. Therefore, $w_{\mathrm{tot}}^{\mathrm{cleared}}=w_{\mathrm{tot}}^{\mathrm{bil}}$, i.e., the total exposure in the system is invariant under central clearing. Notice that the CCP cannot net its trades on its two trade relations because these two are with two different counterparties.

*The perfect non-hedge*: In case *b*_1_ does not hedge, its deal $T\langle b_{1},b_{2}\rangle$ with *b*_3_, but enters into the same deal with *b*_3_, i.e., if it buys $T\langle b_{1},b_{3}\rangle$ instead of $T\langle b_{3},b_{1}\rangle$, then the two deals do not cancel, but add up. In that case, we obtain $w_{\mathrm{tot}}^{\mathrm{cleared}}=2w_{\mathrm{tot}}^{\mathrm{bil}}$ again. This illustrates that the risk in the cleared system depends on the trade relation graph's topology and the trades' netting structure.

We conclude that by flipping the long/short flag of a single trade in that system, the outcome of the central clearing regulation produces a completely different result. This is problematic because the impact of regulations should not depend on such subtleties but should have the desired effect in all reasonable scenarios and even be robust under unlikely scenarios.

### 6.3 The daisy chain

**Figure 6:**
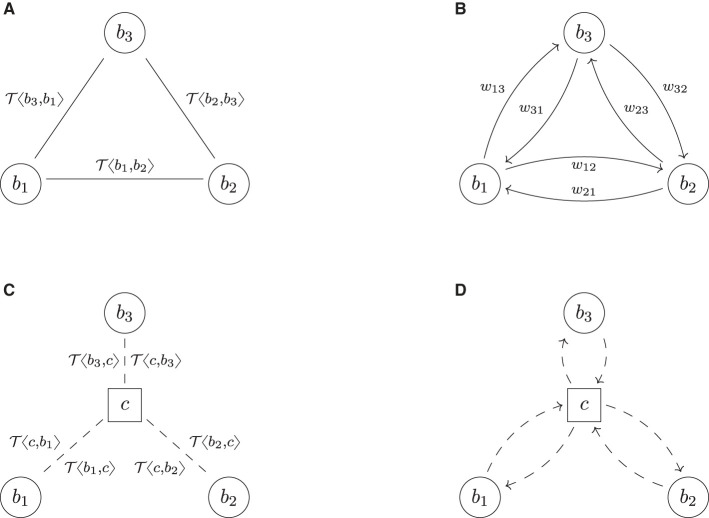
Daisy chain. **(A)** Trade relation graph (bilateral). **(B)** Risk graph (bilateral). **(C)** Trade relation graph (cleared). **(D)** Risk graph (cleared).

We now extend the example in Section 6.2 even further by assuming that *b*_2_ and *b*_3_ both want to hedge their trade with *b*_1_ by entering into the deal $T\langle b_{2},b_{3}\rangle$, thus inadvertently creating a *daisy chain*, see Figures 6A, B.^10^ The three links in these financial systems do not add any economic value and thus could be collapsed. However, this knowledge is not available to any of the three banks in the system as every one of them only has the full knowledge about the trade data attached to the two links connecting it to the system, but not about the third one.

Applying the central clearing operator has a remarkable result shown in Figures 6C, D: all banks have one netting set of two trades, which are perfect hedges of each other, and thus all trade relations are equivalent to winding them down, thus $\bar{w}\left(b_{i}\right)=\bar{w}\left(c\right)=0$, *i* = 1, 2, 3, which implies $w_{\mathrm{tot}}^{\mathrm{cleared}}=0$. This proves that the lower bound in Equation (5) is sharp too.

Notice that in the bilateral system, all banks are perfectly hedged, i.e., they all have zero market risk. However, their credit risk is far from zero: any bank, say *b*_1_, faces the two other banks *b*_2_ and *b*_3_ and is exposed to both their defaults. A default of one of the two would bring the hedge out of balance, and because it cannot be determined *a priori*, one of the two defaults first exposes *b*_1_ to the risk of losing the deal in the money. However, in the cleared system, every bank faces only one counterparty, the CCP, and thus this credit risk vanishes. No counterparty, including the CCP, is exposed to any credit risk.

This example easily generalizes to arbitrary daisy chains, which do not necessarily need to be closed.

**Definition 1 (daisy chain)**. A financial system FS = (*B, L*, τ) is called a *daisy chain* if

(i) The trade relation graph (*B, L*) is simple and a path, i.e., of the form
(12) $$b_{1}\overset{\ell_{1}}{\to }\;b_{2}\;\overset{\ell_{2}}{\to }b_{3}\overset{\ell_{3}}{\to }\ldots \;\overset{\ell_{n-1}}{\to }b_{n},$$
  where *n* = |*B*| and *b*_*i*_≠*b*_*j*_ for 2 ≤ *i, j* ≤ *n*−1.
(ii) For each netting set of trades τ(ℓ_*i*_), there exists a subset *N*_*i*_ such that subsequent subnetting sets of trades are perfect hedges of each other, i.e., *N*_*i*_∪*N*_*i*+1_~**0**.

The daisy chain is *closed* if *b*_1_ = *b*_*n*_.

While a financial system that consists only of a single daisy chain is, of course, a theoretical construct, the real financial system does contain a lot of subgraphs, which are isomorphic to a daisy chain in the sense of Definition 1, and that poses very real practical problems. Daisy chains introduce economically unnecessary amounts of counterparty risk into the system and thus create unnecessary costs to the counterparties to mitigate that risk. Removing daisy chains from a bilateral financial system is a non-trivial problem. It has been observed in Gallagher et al. (2017) that as no bank has the information necessary to detect the existence of a daisy chain, a possible way to tackle this is to anonymously pool risk sensitivities of the counterparties and then perform a constrained optimization procedure to reduce the amount of initial margin resulting from the daisy chains, but keeping the net sensitivities fixed. We observe that the clearing house has all information necessary to detect daisy chains, and the novation of the netting sets as part of the clearing process automatically eliminates all the daisy chains.

Daisy chains are just a particularly extreme case of hedges, and the phenomena observed with daisy chains hold for all perfect hedges.

## 7 Systemic risk simulation engine

The mathematical analysis carried out in the previous sections provides important *a priori* estimates and conceptual insight into the impact of clearing, and the case studies provide illustrations and intuition of these. However, they do not always tell the full quantitative story regarding larger financial systems. Therefore, we now proceed to a case study of larger examples of financial systems using numerical simulation.

**Figure 7:**
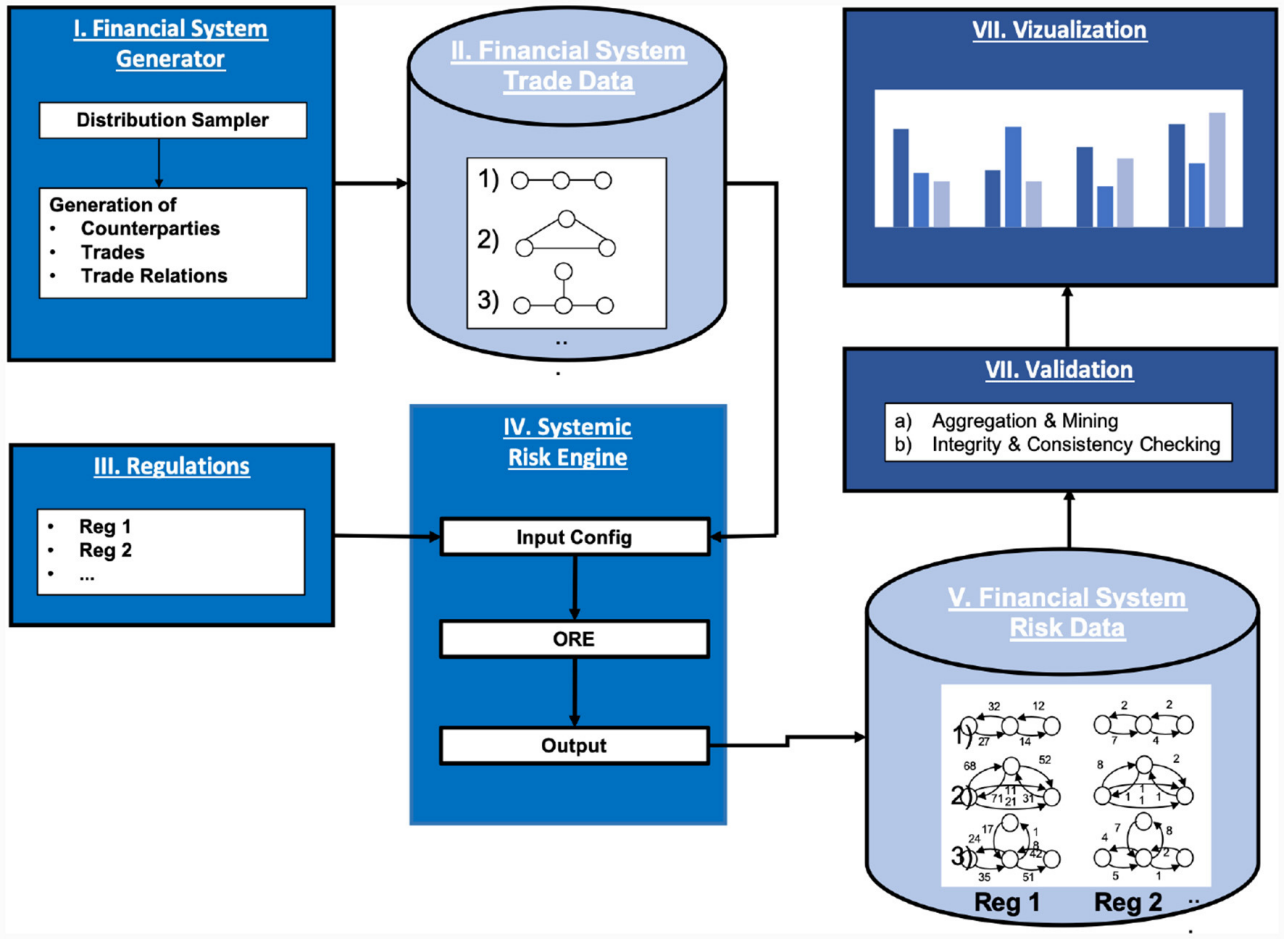
Systemic risk simulation engine.

The high-level concept of the simulation is as follows (see also Figure 7): we first generate a sample set of simulated financial systems, including the trades. Then, under a chosen baseline regulation (bilateral in this case), we compute all the risks generated by all the trades in the financial system and aggregate the outcomes. Finally, we repeat that process for other regulations and compare the results. This effectively tests for each regulation the hypothesis that the regulation makes the system safer than the baseline regulation.

The various steps are described in more detail in the following sections.

### 7.1 Generation of trade relation graph topology

As a first step, we need to generate random trade relation graphs. While the precise trade relations of the actual financial system are confidential, empirical studies based on central bank data on the topology of trade relations graphs are available. These studies show that financial systems always have the property that, on the one hand, there are a few banks at the *periphery* of the graph that only have a few trade relations with the *core*. On the other hand, a few banks in the core have many trade relationships, particularly with each other. This *core-periphery* model can be found, for instance, in Kley et al. (2014) and also in Craig and von Peter (2010), which applies it to the German market. Similar results have been obtained in Cont et al. (2013) for the Brazilian market and in Boss et al. (2004) for the Austrian market—both study the distribution of the node degree of the banks. Both conclude that besides a periphery of banks, which do not have many trade relations, in the core, banks have a Pareto distributed node degree, i.e., there are a few banks with many connections and many banks with fewer connections.

Therefore, for this simulation, we approximate this by assuming that the financial systems have a Pareto distributed node degree (meaning the periphery is a bit more connected and the core a bit less than in the core-periphery model). Generating graphs such that their degree sequence has a prescribed distribution is a non-trivial problem in discrete mathematics. We use the *erased configuration model* as implemented in the Python library networkx to compute the graphs; see also Newman (2003), Britton et al. (2006), and Bayati et al. (2007). The Pareto distributed degree sequences are generated using the Python library numpy.random, see Oliphant (2006). An example of a graph generated with this technique is shown in **Figure 10**, where the Pareto parameters *x*_min_ = 3 and exponent α = 2 are used.

### 7.2 Trade generation

Generating the trade data for the simulation is challenging because the trade deals in the real financial system are strictly confidential and thus cannot be used as an empirical baseline. There are some high-level statistics available, though, for example, in the data warehouse by the BIS^11^ or the annual statistical report by ESMA, see Securities and Authority (2018). These sources provide interesting insights into the financial system, but this data cannot be used to calibrate our simulation because it is not granular enough. The question of how many details about the financial system can be recovered from publicly available data is compelling but also difficult to answer. We plan to investigate this in further research.

**Figure 8:**
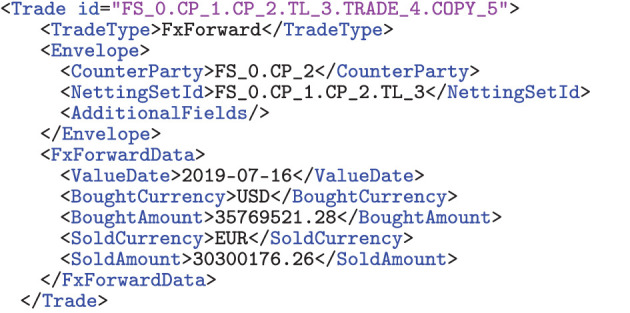
Trade representation in ORE XML.

**Figure 9:**
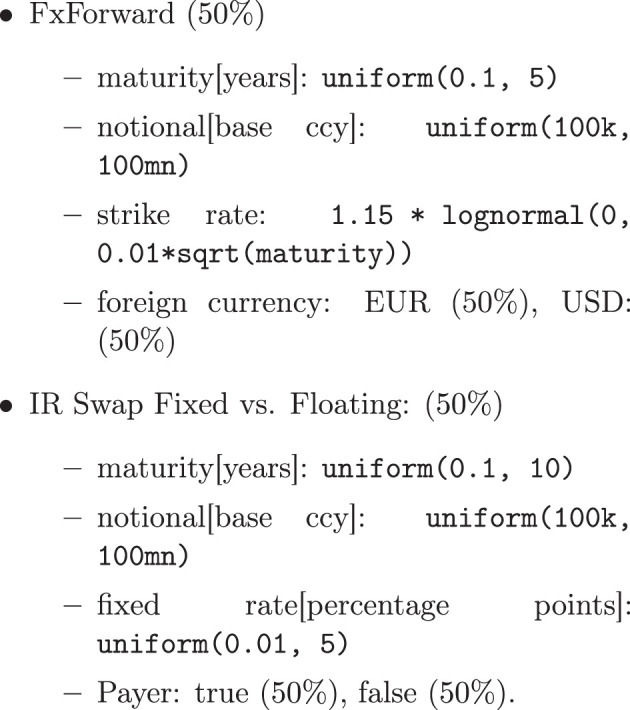
Marginal priors of trade repository generation.

For the simulation at hand, we will choose marginal priors of the trade distributions, see **Figure 9**. While these distributions are arbitrary, one can expect a typical trade in the real financial system to be within their range. We then generate the trade data in two steps: first, we create a repository of trades using the marginal priors. Second, we assign these trades to trade relations in the financial system by choosing a uniformly distributed number *k* between a lower and an upper bound for each link in the system. The trade list on that link is then filled by simply drawing from the generated repository with replacement (for any given link), where all trades are chosen with equal probability. The results are converted into ORE XML format, such as the example shown in Figure 8 using the Python library lxml.

### 7.3 Counterparty risk simulation

In the next step, we need to compute the risks in every trade of every netting set from the perspective of every bank under every regulation. Even calculating the risks in one derivative from the perspective of one bank is a non-trivial task, which typically falls into the responsibility of a bank's risk function. In theory, one could use the risk management system of any bank for that purpose, but the code of these proprietary systems is as confidential as the trade data that flows through them. Consequently, these engines are not available for academic research. Therefore, we use the *Open Source Risk Engine (ORE)* instead, see ORE. The reason for this choice is that its license allows the use for academic research, its design is of a similar style as proprietary risk engines and due to the open source model its calculations are transparent, which enables validation and replication.

ORE consumes a derivatives portfolio with netting information, market data, and various configuration files and computes the risks in that portfolio from the perspective of a single bank. The risks are calculated by first identifying and evolving the relevant risk factors of a portfolio, such as interest and FX rates. ORE then simulates these risk factors into the future with risk factor evolution models calibrated to a current market data snapshot. The cross-asset simulation rests on a 1-factor Hull-White model for the interest rate and a geometric Brownian motion for the FX rate. These models introduced in Hagan (2015), and Hull and White (1990) have the advantage that they are very well established. The evolved risk factors are then used to price the derivatives using risk-neutral valuation and compute exposures and XVAs. Finally, the results are aggregated into standard regulatory exposure metrics. More background on the methodology can be found in Lichters et al. (2015). Technically, ORE is implemented in C++ and rests on QuantLib, see QuantLib. More details on ORE's data flow and the input/output formats can also be found in its user guide; see ORE (2018).

### 7.4 Aggregation

A key output of the ORE simulation is a set of csv files containing the risk metrics of the input trades. We use the Python library pandas to parse these files and then compute the risk graphs of all the trade relation graphs in the system (including all the weights). We then aggregate this information to the counterparty level and then to the systemic level. Finally, we compute a comparison data frame to compare the results under different regulatory regimes. Notice that this data flow mirrors the level of aggregation when passing from a microeconomic view of a financial system to a macroeconomic view—hence bridging the gap between the two.

### 7.5 Validation

The inputs and outputs of a standard ORE configuration are already quite complex. The configurations we used to model the entire financial systems under multiple regulations and produce input configurations and output files which are too big to be human-readable and have a very complex structure. To validate the intermediate and final results, we perform integrity and consistency checks on the input XML files, the output csv files, and the trade relationship and risk graphs. These checks include, for example, that the NPV of every trade has the opposite sign for the two counterparties, we check that the number and id of deals, counterparties, and systems are consistent across the various files, and we also check that the impacts guaranteed by Theorem 1 hold. Technically, we perform these tests using the Python library unittest. Using unit tests to test data rather than code is a rather novel technique dubbed TDDA (test-driven data analysis).^12^

### 7.6 Visualization

To inspect, understand and visualize the outputs, we use a jupyter notebook to create various views on the data using ipywidgets to produce interactive plots drawn using matplotlib. This notebook has produced all the results shown in Section 8, for example, **Figure 11**.

## 8 Simulation case study results

In this section, we present the results^13^ obtained by running the systemic risk engine described in Section 7.

### 8.1 Simulation setup

**Figure 10:**
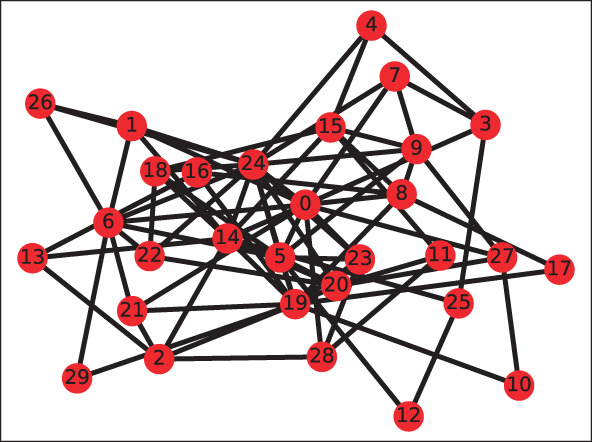
Example of a trade relation graph, where the node degrees are sampled from a Pareto distribution with *x*_min_ = 2, α = 2.

We generate a repository of 10, 000 trades according to the logic described in Figure 9 and 2 financial systems^14^ with 30 counterparties each. The trade links are created according to the logic described in Section 7.1 with a minimum of *x*_*m*_ = 3 and a Pareto exponent of α = 2.^15^ The links are then populated by randomly choosing a uniformly distributed number of 5–15 trades from the trade repository. The systems have a total of 2, 988 trades in 294 netting sets, see Figure 10 for a visualization of a trade relation graph.

For the ORE simulations, we need to supply a full set of inputs, which besides the trade and netting relations described above, also need a market data snapshot that contains, in particular, the implied volatilities and mean reversions for the interest rate model, the implied volatilities for the FX model and the instantaneous correlations between all these risk factors. Obtaining consistent and up-to-date market data for such simulations is a non-trivial and cost-intensive problem that most banks solve by entering into suitable licensing agreements with their market data vendors. For the purpose of this case study, we use the free market data snapshot for 05/02/2016 that is supplied with the engine.^16^ For the curve conventions and simulation settings, we use the standard config.^17^ The number of Monte Carlo paths is set to 1, 000.

We then define the six regulations corresponding to the formalizations in Section 4, namely bilateral, pre_cleared, cleared, repartitioned, multi_pre_cleared and multi_cleared and study their impact measured in EEPE and CVA. For central clearing, only *m* = 1 clearing house is used; for multi-clearing, *m* = 2 clearing houses are used. The impact measured in CVA depends on the rating of the CCPs. We avoid the problems with determining the CVA of the CCP discussed in Section 5.4 by mandating the rating of the CCPs: we use the three fictitious ratings^18^ AA, AAA, and AAAA, which we arbitrarily define by flat hazard rate of 2%, 1% and 0.5%. We assume that all banks in the system have a rating of AAA. We then study the six base regulations under three different ratings of the clearing house. As bilateral and repartitioned do not depend on the rating, we obtain a total of 14 regulations, including bilateral, which serves as our baseline.

### 8.2 Average impact of regulations

**Figure 11:**
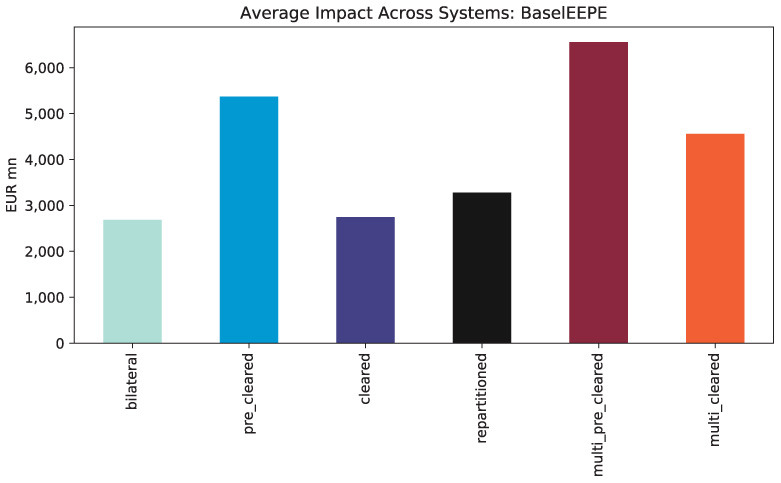
Average impacts across systems (EEPE).

The average total impact of the regulations on the financial systems measured in EEPE is shown in Figure 11. We find that pre-clearing doubles the exposure while clearing then reduces it again—in this case, to nearly the same level as before. Thus, central clearing has almost no effect in this case. Multi-clearing through *m* = 2 CCPs has an adverse effect as the repartitioning increases the exposures further. Notice that these results align with the *a priori* estimates Theorem 1.

**Figure 12:**
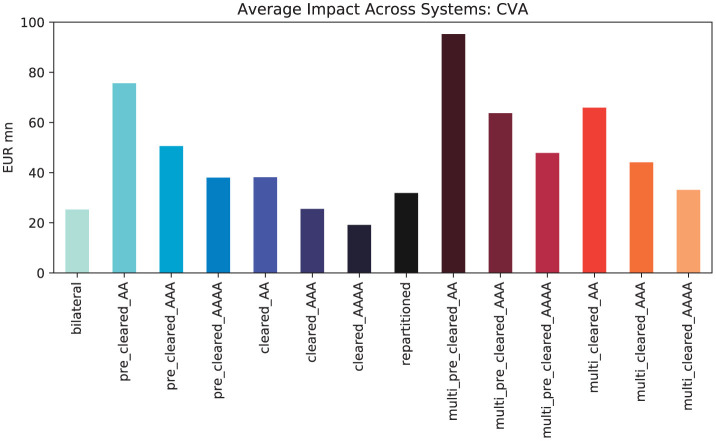
Average impacts across systems (CVA): comparison of clearing regulations against bilateral baseline in low (AAAA), medium (AAA) and high (AA) risk scenarios.

When measuring the effect in CVA, the situation becomes significantly more complex; see Figure 12. For the AAA rating, the impact of clearing measured in CVA is qualitatively the same as measured in EEPE because, in that case, all counterparties in the system have the same PD, and all exposures are weighted equally. The adverse effects of clearing are exacerbated when the credit quality of the CCPs deteriorates, i.e., in the AA rating. For the maximum rating AAAA, we can see that the centrally cleared system is now finally safer than the bilateral system. But even when both CCPs are AAAA-rated, the risk in the multi-cleared system is still higher than in the bilateral system. For all regulations, the risk is monotonously decreasing in the rating, i.e., the risk gets lower when the rating for the CCPs improves, which is in line with intuition. The conclusion is that the more CCPs are used for multi-clearing, the lower their PD has to be to obtain a multi-cleared system, which is safer than the bilateral one.

### 8.3 Impact on banks

**Figure 13:**
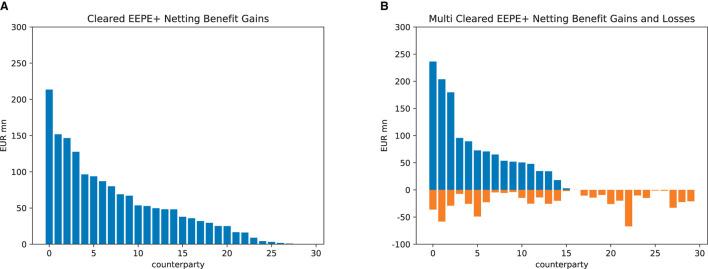
Absolute netting benefits (EEPE). **(A)** Central clearing. **(B)** Multi-clearing.

We now drill into the first financial system (the impacts on the other one are similar) and study the impact of the regulations on the various banks. In Section 5, we identified the netting benefits as the decisive factor for these. They cannot be determined *a priori* but can be computed in the simulation; see Figure 13. In the case of central clearing, see Figure 13A, all banks enjoy exposure netting benefits, and thus, central clearing is unambiguously risk-reducing for the banks (however, the sum of these netting benefits may or may not outweigh the additional risks introduced by the clearing house). In Figure 13B, we see that in the case of multi-clearing, the netting benefit gains are accompanied by netting benefit losses. Thus the total impact on a given bank may reduce or increase its exposures. We conclude that it cannot be guaranteed that multi-clearing has the desired effect.

**Figure 14:**
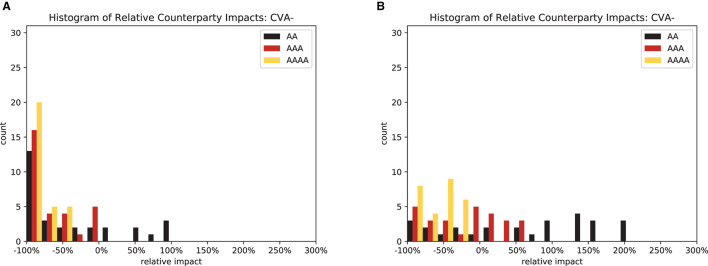
Relative counterparty impacts (CVA−). **(A)** Central clearing. **(B)** Multi-clearing.

In particular, for CVA, it is instructive to compute a histogram of the relative impact of the regulations, sees Figure 14. We observe two effects: first, for both central and multi-clearing, the deteriorating credit quality of the CCPs makes the distribution of impacts wider and more skewed toward risk increases. Second, passing from central clearing, see Figure 14A, to multi-clearing, see Figure 14B, has the same effect. This means that the more CCPs are in use, the better their credit quality will be to have the desired effect.

### 8.4 Risk concentrations

**Figure 15:**
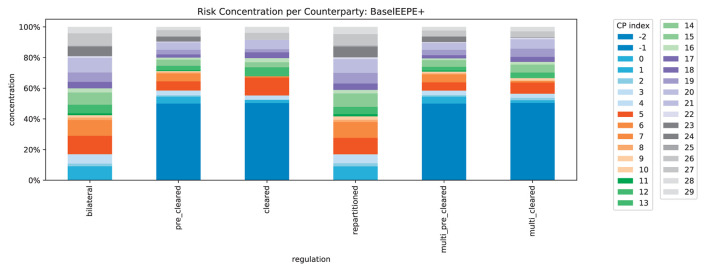
Relative impacts per counterparty in system (EEPE+).

**Figure 16:**
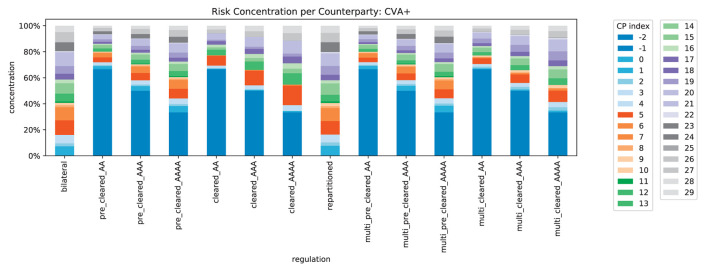
Relative impacts per counterparty in system (CVA+).

We compute the concentration of risk exposures in the system, recall Equation (4), for all counterparties, see Figure 15. In line with Corollary 5, we find that exactly 50% of the exposure is concentrated in the CCPs (indexed with −1 and −2 here). The risk concentration of the original banks is squeezed into the remaining 50%. In Figure 16, we can see that this no longer holds for CVA^+^: when the credit quality of the CCPs deteriorates, the risk is even more concentrated in the CCPs. This illustrates how strongly the safety and soundness of the system depend on the credit quality of the CCPs.

## 9 Conclusion

We have presented an approach to analyze the impact of regulation on systemic risk by enhancing the standard risk graph model to include an underlying trade relation graph. This enables three forms of analysis: (i) theoretically motivated a prioi estimates, (ii) stylized case studies, and (iii) simulation case studies.

**Figure 17:**
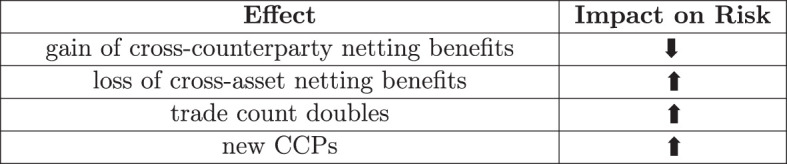
Competing effects.

In the case of central clearing, we have shown how the *a priori* analysis of the graph models yields insights into the total levels and concentration of exposure and have identified the netting benefits and losses as a key driver impacting systemic risk. The stylized case studies of even very simplified financial systems provide a clear illustration on how the *a priori* estimates can play out under various scenarios. Finally, we have shown that it is technically feasible to create a simulation environment for a much more sophisticated case study via a systemic risk engine. While the proprietary trade data of the real financial system is not available, we have shown that this is sufficiently robust to deliver front-to-back analysis of entire financial systems that have synthetic trade data. Even with limited computational resources and hence limited samples size of 10 financial systems, it yields detailed micro- to macro insights on how the regulation plays out. Not only do we obtain the total average impacts on exposure, CVA and concentration, but also detailed visibility of the netting benefits and losses on a bank level. The disentanglement of the competing effects driving the impact of central clearing is summarized in Figure 17. Depending on the various magnitudes, central clearing can either make a positive or a negative impact on the risks in the system as a whole. The benefit is that regulators or policymakers can now identify more precisely potential points of failure of regulation and alter the regulation accordingly. In this case, an example would be the concentration risks and the adverse effects of too many clearing houses.

While the simulation case study is much more sophisticated and realistic than the stylized case studies, it nevertheless cannot capture all possible aspects. Nonetheless, the flexibility of our method allows the analysis to expand in multiple directions:

Calibration: in the simulation described in Section 7, we generate the trade distribution using the marginal priors summarized in Figure 9. Publicly available statistics such as the ESMA report, see Securities and Authority (2018), or the BIS website (see text footnote 11) indicate that the marginal priors used in our simulations capture typical trades. These priors can be refined such that the distributions of trade features in the simulated system match the analogous distributions in empirical data more closely.
Robustness: it is neither possible to calibrate the simulation to that empiric data uniquely nor is it advisable as the impact of regulation should not be overly sensitive to the chosen trade data. Our framework enables us to conduct robustness tests of the underlying assumptions by varying the distributions that generate the trade data.
Agent-based modeling: in the next step, one could remove the marginal priors and instead use an agent-based modeling approach on the nodes, for which the resulting trade distributions are an output rather than an input to the simulation. Agent-based modeling has been used in systemic risk research, for example, in housing market studies, see Geanakoplos et al. (2012).
Correlations: we have pointed out in Section 3.5 that the logic to aggregate risks from the links to the nodes and the system implicitly assumes a maximally adverse correlation structure of the risks. This is a business practice and regulatory standard for aggregating exposures, but this picture of the correlations is inaccurate. We plan to estimate a correlation structure in the simulation and incorporate it into the risk metrics.
Liquidity: the metrics in the risk graph can measure arbitrary types of risk, yet we have only used EEPE and CVA to measure counterparty credit risk. However, liquidity risk was one of the most dangerous types of risk in the aftermath of the financial crisis. Therefore, we plan to add liquidity risk metrics to the simulation to analyze the impact along this additional dimension.
Collateralization: the present paper studies the impact of central clearing without collateralization, and we have studied the impact of collateralization without clearing, see O'Halloran and Nowaczyk (2019). We plan to study the combined impacts, particularly the above-mentioned liquidity risks.
Cloud: finally, we plan to scale up the magnitude of the simulation by using cloud-based technology.

## Data availability statement

The original contributions presented in the study are included in the article/Supplementary material, further inquiries can be directed to the corresponding authors.

## Author contributions

All authors listed have made a substantial, direct, and intellectual contribution to the work and approved it for publication.

## Footnotes

^1^See O'Halloran and Groll (2019) for a review of government responses to the 2008–2009 financial crisis.

^2^From a financial economics perspective, one can alternatively think of this hypothesis testing as being derived from an agent-based model, where the agent is the regulator. This agent is risk-adverse and thus tries to minimize the firm exposures and the aggregate levels of risk in the system by changing financial regulation under the constraint that the cashflows of all trades remain constant.

^3^In a multigraph, any two nodes might be connected with more than one link. Consequently, the links between two nodes might be a multiset rather than a set.

^4^Notice that this formalism can be easily modified and extended by changing the equivalence relation to incorporate other features of the deal, for instance, the notional or the currency (we do neither need nor use this in the present study though).

^5^In summary, these metrics are defined as follows: if *V*(*t*) denotes the simulated portfolio value at *t* (possibly net of collateral), then we first define the *Expected Positive Exposure* EPE(*t*): = E[*V*(*t*)^+^]. This value is then first effectivized via $\text{EEE}\left(t\right):={\text{max}}_{0\leq s\leq t}\text{EPE}\left(s\right)$ and finally integrated to $\mathrm{EEPE}:=\frac{1}{T^{\prime }}\int_{0}^{T^{\prime }}\mathrm{EEE}\left(s\right)ds$, where *T*′ = min(*T*, 1*Y*) is the maturity of the portfolio capped at 1*Y*. The CVA is defined by $CVA:=\int_{0}^{T}𝔼\left[D\left(s\right)V{\left(s\right)}^{+}\right]\mathrm{PD}\left(s\right)ds$, where PD is the probability of default of the counterparty and *D* denotes the discount factor.

^6^This has counter-intuitive consequences in cases where the correlation is −1. This case occurs when a bank *b* hedges a netting set *s*_1_ of deals with a bank *b*_1_ with a netting set *s*_2_ with a bank *b*_2_. While both counterparties *b*_1_ and *b*_2_ can default separately or simultaneously, it cannot happen that both default and both netting sets are in the money. Nevertheless, *w*(*b*) is the sum of the risks in *s*_1_ and *s*_2_.

^7^Mathematically, this means that the trade relation graph is *simple*.

^8^recall the definition Equation (3).

^9^For this text, we assume that all derivatives are hedged with other derivatives, which thus also need to be cleared.

^10^See Gallagher et al. (2017) for a discussion on the optimal bilateral derivatives.

^11^See https://www.bis.org/statistics/dataportal/derivatives.htm.

^12^See http://www.tdda.info/ for a library implementing this specifically for anomaly detection.

^13^The underlying Jupyter notebook is available here: https://github.com/niknow/systemic-risk.

^14^For a given effort of computation, one can either increase the number of financial systems or the number of counterparties within each system. Increasing the number of counterparties yields more interesting insights than generating more financial systems sampled from the same distributions.

^15^The reason for this choice of parameters is that these result in a good trade-off: on the one hand the resulting trade relation graphs still qualitatively exhibit the core-periphery property as evident from Figure 10, i.e., the core has many nodes that are connected to many other nodes in the core, but at the periphery the node degree is lower. On the other hand, the total number of nodes and links is small enough such that the total computational effort is still manageable for us to run.

^16^ https://github.com/OpenSourceRisk/Engine/blob/v1.8.4.0/Examples/Input/market_20160205_flat.txt

^17^See https://github.com/OpenSourceRisk/Engine/blob/v1.8.4.0/Examples/Input.

^18^We only use these ratings as scenario labels. In particular, we do not claim that any of the big rating agencies would assign these ratings given the chosen hazard rates in practice.
