# Supplementary material for: Computing the impact of central clearing on systemic risk
Source: Front Artif Intell. 2024 Feb 21;7:1138611. doi: 10.3389/frai.2024.1138611 (PMC10915250; doi:10.3389/frai.2024.1138611)
Supplement: Supplementary file 1 [file Data_Sheet_1.pdf]

# A Appendix

## A.1 Graph Theory Basics

We recall some basic notions in graph theory and introduce formal notation. More on graph theory, particularly spectral graph theory, can be found in standard textbooks.[20]

**Definition A.1** (multiset)**.** A *multiset* (of finite multiplicity) is a tuple $\mathfrak{L} = (L, \mu_L)$, where $L$ is a set and $\mu_L : L \to \mathbb{N}_{\geqslant 1}$ is a function associating each element $\ell \in L$ its *multiplicity* $\mu_L(\ell)$. The set

$$\mathscr{L} := \bigcup_{\ell \in L} \bigcup_{i=1}^{\mu_l(i)} (l, i)$$

is called the *set representation of* $\mathfrak{L}$. A multiset is *simple*, if $\mu_L \equiv 1$. ◊

We will identify a multiset by its set representation and a simple multiset $\mathfrak{L} = (L, \mu_L)$ by its underlying set $L$. The tuple $(l, i)$ is also denoted by $l_i$ sometimes.

**Definition A.2** (undirected multi graph)**.** An *undirected multi graph* is a tuple $G = (V, E)$, where the *vertices* $V$ are a set and the *edges* $E$ are a multiset of two-element subsets of $V$. A multigraph is called *simple* (or just a *graph*) if $E$ is simple. Two vertices $v_1, v_2 \in V$ are *adjacent*, $v_1 \sim v_2$, if $\{v_1, v_2\} \in E$. For any vertex $v \in V$, we define the set

$$V(v) := \{v' \in V \mid v \sim v'\} \tag{A.1}$$

of adjacent vertices and the set of all edges

$$E(v) := \{e \in E \mid v \in e\} \tag{A.2}$$

connecting $v$ to its adjacent vertices. ◊

**Definition A.3** (connected)**.** A multigraph $G = (V, E)$ is *connected*, if for any two distinct vertices $v, v' \in V$ there exists a sequence of vertices $v_1, \ldots, v_k \in V$ such that $v_1 = v$, $v_i \sim v_{i+1}$ and $v_k = v'$. ◊

**Definition A.4** (directed multigraph)**.** A *directed multi graph* is a tuple $G = (V, A)$, where the *vertices* $V$ are a set, and the *arrows* $A$ are a multiset of two-tuples of elements in $V$. A directed multigraph is *simple* if $A$ is a set. Two vertices $v_1, v_2 \in V$ are *adjacent*, $v_1 \sim v_2$, if $(v_1, v_2) \in E$. For any vertex $v \in V$,

[20] for example: Chung. *Spectral Graph Theory.* American Mathematical Society (1997).

we define the multisets

$$V^+(v) := \{v' \in V \mid v \sim v'\}, \tag{A.3}$$

$$V^-(v) := \{v' \in V \mid v' \sim v\} \tag{A.4}$$

of adjacent outgoing and incoming vertices and the multisets of all arrows

$$A^+(v) := \{(v, v') \mid (v, v') \in A\}, \tag{A.5}$$

$$A^-(v) := \{(v', v) \mid (v', v) \in A\} \tag{A.6}$$

connecting $v$ to its adjacent vertices. The same notions apply to simple directed graphs, and then the multisets $V^{\pm}(v)$ and $A^{\pm}(v)$ are sets. ◊

**Definition A.5** (weighted out/in-degree)**.** Let $G = (V, A)$ be a directed multigraph and $w : A \to \mathbb{R}$ be any function. For each vertex $v \in V$, the quantities

$$w^{\pm}(v) := \sum_{a \in A^{\pm}(v)} w(a) \tag{A.7}$$

are called the *weighted out/in degree*. Let $w(G) := \sum_{a \in A} w(a)$ be the total weight in the system. The quantities

$$\rho^{\pm}(v) := \frac{w^{\pm}(v)}{w(G)}, \tag{A.8}$$

are called *weighted relative out/in-degree.* In case the function $w : A \to \mathbb{R}^k$ is multivariate, the weighted in/out degree $w : V \to \mathbb{R}^k$ and the relative weighted in/out-degree $\rho : V \to \mathbb{R}^k$ are defined using Eqs. (A.7) and (A.8) in each component. ◊

Notice that in case $k = 1$ and $w \equiv 1$, we have $w^{\pm}(v) = \deg^{\pm}(v)$, i.e., the weighted in/out-degree is the ordinary in/out degree, which just counts the number of incoming respectively outgoing edges.

**Theorem A.6** (spectrum of the star)**.** The star-shaped graph with $n$ nodes has the spectrum

$$\{0, \pm\sqrt{n-1}\}, \tag{A.9}$$

where $\pm\sqrt{n-1}$ both have multiplicity 1 and 0 has multiplicity $n - 2$. ◊

**Proof.** Assuming that the center of the graph is node $i = 1$, the adjacency matrix $A_n$ of the star is

given as

$$A_n := \begin{pmatrix} 0 & \mathbf{1}_{n-1}^t \\ \mathbf{1}_{n-1} & \mathbf{0}_{n-1} \end{pmatrix} \in \mathbb{R}^{n \times n},$$

where $\mathbf{0}_n \in \mathbb{R}^{n \times n}$ is the zero matrix and $\mathbf{1}_n \in \mathbb{R}^{n \times 1}$ is a vector of 1. We prove the following via induction over $n$:

$$\chi_n := \det(A_n - X\mathbf{I}_n) = (-1)^n (X^n - (n-1)X^{n-2}). \tag{A.10}$$

For $n = 2$, we obtain

$$\chi_2 = \det(A_2 - X\mathbf{I}_2) = \begin{vmatrix} -X & 1 \\ 1 & -X \end{vmatrix} = X^2 - 1.$$

For $n - 1 \to n$, we use the Laplace expansion on the $n$-th row to conclude

$$\begin{aligned} \chi_n = \det(A_n - X\mathbf{I}_n) &= \begin{vmatrix} -X & \mathbf{1}_{n-1}^t \\ \mathbf{1}_{n-1} & -X\mathbf{I}_{n-1} \end{vmatrix}, \\ &= (-1)^{n+1}(-1)^{n-1+1} \det(-XI_{n-2}) \\ &\quad + (-1)^{n+n}(-X)\chi_{n-1} \\ &= (-1)^{n+1}X^{n-2} + (-1)^n X(X^{n-1} - (n-2)X^{n-3}) \\ &= (-1)^n (X^n - (n-1)X^{n-2}). \end{aligned}$$

Finally, the factorization

$$\chi_n = (-1)^n X^{n-2}(X^2 - (n-1))$$

implies the claim. □

## A.2 Mathematics of the Graph Model

For any set $Y$, we denote by $[Y]$ the set of all finite subsets of $Y$.

**Definition A.7** (financial system)**.** A *financial system* $\mathrm{FS} = (B, \mathscr{L}, \tau)$ is an undirected multigraph $G = (B, \mathscr{L})$ called *trade relation graph* together with a *trade data function* $\tau : \mathscr{L} \to [Y]$. Optionally,

this can be enriched by a *node data function*[21] $\beta : B \to X$. ◊

**Definition A.8** (risk graph)**.** Let $\mathrm{FS} = (B, \mathscr{L}, \tau, \beta)$ be a financial system as in Definition A.7. A weighted directed multigraph $\mathrm{RG} = \mathrm{RG}(\mathrm{FS}) = (B, \mathscr{A}, w)$ is called a *risk graph associated to* FS, if

(i) $B$ is the set of nodes in the graph representing the banks (i.e., FS and RG have identical nodes).

(ii) $\mathscr{A}$ is the multiset of arrows (directed edges) in the graph RG. For each trade relation $\ell = \{b_1, b_2\}$ between a bank $b_1 \in B$ and $b_2 \in B$, the multiset $\mathscr{A}$ contains two arrows $(b_1, b_2), (b_2, b_1) \in \mathscr{A}$ in opposite directions, i.e.

$$\mathscr{A} = \bigcup_{\{b_1,b_2\}\in\mathscr{L}} \{(b_1, b_2), (b_2, b_1)\}. \tag{A.11}$$

as multisets.

(iii) $w : \mathscr{A} \to \mathbb{R}^k$ is a multivariate weight function on the arrows quantifying the risks attached to each arrow measured in $k$ metrics.[22] ◊

**Lemma A.9.** Let $\mathrm{FS} = (B, \mathscr{L}, \tau)$ be a financial system and $\mathrm{RG} = (B, \mathscr{A}, w)$ be an associated risk graph. Then the degree $\deg(b)$ of any node $b \in B$ in FS is related to its out/in-degree in RG via:

$$\forall b \in B : \deg(b) = \deg^{\pm}(b). \tag{A.12}$$

◊

**Proof.** This follows directly from Definitions A.7 and A.8 and the definition of a degree. □

**Definition A.10** (risk aggregation)**.** Let $\mathrm{RG} = (B, \mathscr{A}, w)$ be a risk graph as in Definition A.8. For any $b \in B$, we define the risk induced/received by

$$w^{\pm}(b) = \sum_{\ell\in\mathscr{A}^{\pm}(b)} w(\ell) \tag{A.13}$$

and the total level of risk by

$$w(G) := \sum_{b\in B} w^{+}(b). \tag{A.14}$$

[21] and, in theory, any additional static data.

[22] While the space $\mathbb{R}^k$ is sufficient for all numerical applications, it is mathematically possible to allow a metric to be a function $\mathbb{R}_{\geqslant 0} \to \mathbb{R}^k$ or even a stochastic process $\Omega \to \mathbb{R}^k$ .

We define the relative risks induced/received by any node by

$$\rho^{\pm}(b) = \frac{w^{\pm}(b)}{w(G)}. \tag{A.15}$$

◊

We recall the following definition from [26]:

**Definition A.11** (systemic risk)**.** Let RG $= (B, \mathscr{A}, w)$ be a risk graph as in Definition A.8. Any of the quantities

$$w(\mathrm{RG}), \qquad \max_{b \in B} w^{\pm}(v), \qquad \max_{v \in V} \rho^{\pm}(v) \tag{A.16}$$

are regarded as a *metric of systemic risk.* ◊

We denote by $\mathbf{0}$ any netting set that does not generate any cashflows.

## A.3 Risk Metrics

**Definition A.12** (bilateral risk metric)**.** Let FS $= (B, \mathscr{L}, \tau)$ be a financial system. A *bilateral risk metric* is a function $w$ such that for any trade relation $\{b_1, b_2\}_i \in \mathscr{L}$ and any two possible netting sets $s_1 = \tau_1(\{b_1, b_2\}_i)$, $s_2 = \tau_2(\{b_1, b_2\}_i)$ of trades between them, the following hold:

(i) $w(s_1) \geqslant 0$, i.e. $w$ is a function $w : \mathscr{A} \to \mathbb{R}_{\geqslant 0}$,

(ii) $w(\mathbf{0}) = 0$, i.e. no trades yield to no risk,

(iii) $w(s_1 \cup s_2) \leqslant w(s_1) + w(s_2)$, i.e. $w$ is monotonous with respect to netting benefits.

A bilateral risk metric is an *exposure metric* if $w(s_1)$ is independent of $b_1$ and $b_2$. ◊

**Remark A.13** (standard exposure metrics)**.** Examples of exposure metrics in the sense of Definition A.12 include[23]:

(i) $V(t, \omega)^{\pm}$: The positive/negative part of the simulated value $V$ of the netting set at time $t > 0$ in a realization $\omega$.

(ii) EPE($t$): The *Expected Positive Exposure* at any time $t > 0$.

(iii) ENE($t$): The *Expected Negative Exposure* at any time $t > 0$.

(iv) $\mathrm{PFE}_{\alpha}(t)$: The *Potential Future Exposure* at time $t > 0$ and confidence level $\alpha$.

[23] The precise mathematical formulation of these metrics, which we are using, is described in the user guide of the open risk engine, see [37, Appendix A.3].

(v) EEPE The *Effectivized Expected Positive Exposure.*

(vi) $\mathrm{NPV}^{\pm}$ the positive/negative part of the current *Net Present Value* of the netting set. ◊

## A.4 Mathematics of Clearing Operators

**Definition A.14** (repartitioning). Let $\mathrm{FS} = (B, \mathscr{L}, \tau)$ be a financial system with trade data function $\tau : \mathscr{L} \to [Y]$, $\Gamma = \{\gamma_1, \ldots, \gamma_m\}$ be a finite set and $\alpha : Y \to \Gamma$ be any function associating to any trade $y \in Y$ its *class* $\gamma(y) \in \Gamma$. Then $\mathrm{RP}_\alpha(\mathrm{FS}) := \breve{\mathrm{FS}} := (\check{B}, \check{\mathscr{L}}, \check{\tau})$, is called the *$\alpha$-repartitioning of* FS, where

- $\check{B} := B$, i.e. the nodes are preserved.
- For each link $\ell \in \mathscr{L}$, there is precisely one link $\ell_\mu \in \check{\mathscr{L}}$ parallel to $\ell$ for each class $\gamma_\mu$ that occurs in the netting set $\tau(\ell)$,
- $\check{\tau}(\ell_\mu) := \alpha|_{\tau(\ell)}^{-1}(\gamma_\mu)$, i.e. all the trades in $\tau(\ell)$ of class $\gamma_\mu$ are attached to $\ell_\mu$.

We say that FS is *$\alpha$-partitioned* if every netting set $\tau(\ell)$ consists of only one class, i.e. $|\alpha(\tau(\ell))| = 1$.◊

**Lemma A.15** (properties of $\alpha$-repartitioning). Using the notation of Definition A.14, the following hold:

(i) $\mathrm{RP}_\alpha(\mathrm{FS})$ is $\alpha$-partitioned.

(ii) $\mathrm{RP}_\alpha$ is *idempotent*, i.e. $\mathrm{RP}_\alpha^2 = \mathrm{RP}_\alpha$.

(iii) If $m = |\Gamma| = 1$, then $\mathrm{RP}_\alpha(\mathrm{FS}) = \mathrm{FS}$, i.e., $\mathrm{RP}_\alpha$ is the identity operator.[24] ◊

**Definition A.16** ($m$-pre-clearing operator). Let $\mathrm{FS} = (B, \mathscr{L}, \tau)$ be a trade relation graph with trade data function $\tau : \mathscr{L} \to [Y]$. Assume that $\alpha : Y \to \Gamma$, $\Gamma = \{\gamma_1, \ldots, \gamma_m\}$, is a function and that FS is $\alpha$-partitioned. Then the *$m$-pre-clearing operator* $\widehat{\mathcal{MC}}^{(m)}$ *with respect to* $\alpha$ associates to each such trade relation graph FS a trade relation graph $\widehat{\mathrm{FS}}^{(m)} = (\hat{B}, \hat{\mathscr{L}}, \hat{\tau})$, called the *$m$-pre-clearing of* FS, as follows:

(i) $\hat{B} := B \dot\sqcup \{c_1, \ldots, c_m\}$, i.e. the nodes in $\hat{\mathrm{FS}}^{(m)}$ are the same as in FS with $m$ new nodes $c_1, \ldots, c_m$ added, called *Central Counterparties (CCPs).*

[24] For that reason, this step is unnecessary and irrelevant for central clearing.

(ii) $\hat{\mathscr{L}}$ is given as follows: As FS is $\alpha$-partitioned, for each link $\ell = \{b_1, b_2\}_i \in \mathscr{L}$, we have $\alpha(\tau(\ell)) = \{\gamma_\mu\}$. In $\hat{\mathscr{L}}$ this link is broken up and $c(\ell) := c_\mu$ is inserted, i.e. there exist precisely two links $\ell_1 := \{b_1, c_\mu\}, \ell_2 := \{c_\mu, b_2\} \in \hat{\mathscr{L}}$ for each such link $\ell \in \mathscr{L}$. Formally,

$$\hat{\mathscr{L}} := \bigcup_{\ell=\{b_1,b_2\}\in\mathscr{L}} \{\{b_1, c(\ell)\}, \{c(\ell), b_2\}\} \tag{A.17}$$

as a multiset.

(iii) The function $\hat{\tau}$ is defined as follows: For each link $\ell = \{b_1, b_2\}_i \in \mathscr{L}$ corresponding to the two links $\ell_1 = \{b_1, c_\mu\}, \ell_2 = \{c_\mu, b_2\} \in \hat{\mathscr{L}}$ as above, we define $\hat{\tau}(\ell_1) := \alpha|^{-1}_{\tau(\ell)}(\gamma_\mu)$, where in each trade, the side $b_2$ is replaced by $c_\mu$. That means we attach to $\ell_1$ all trades in $\tau(l)$ of class $\gamma_\mu$. The same is applied vice versa to $\ell_2$.[25]

In case FS is not $\alpha$-partitioned, its $m$-pre-clearing is defined as the $m$-pre-clearing of its $\alpha$-repartitioning, i.e.

$$\widehat{\mathcal{MC}}^{(m)}(\mathrm{FS}) := \widehat{\mathcal{MC}}^{(m)}(\mathrm{RP}_\alpha(\mathrm{FS})).$$

In case $m \geqslant 2$, we call $\widehat{\mathcal{MC}}^{(m)}$ *multi-pre-clearing* and in case $m = 1$, we call $\widehat{\mathcal{CC}} := \widehat{\mathcal{MC}}^{(1)}$ *central pre-clearing* and set $\widehat{\mathrm{FS}} := \widehat{\mathrm{FS}}^{(1)}$. ♢

**Remark A.17** (central pre-clearing)**.** In case of a single CCP $c = c_1$, Definition A.16 can be slightly simplified: The nodes are then given as $\hat{B} = B \dot\cup \{c\}$, the links are given by

$$\hat{\mathscr{L}} := \bigcup_{\{b_1,b_2\}\in\mathcal{L}} \{\{b_1, c\}, \{c, b_2\}\}, \tag{A.18}$$

and the function $\hat{\tau}$ is defined as follows: For each link $\{b, c\}_i \in \hat{\mathscr{L}}$ corresponding to a link $\{b, b'\}_j \in \mathscr{L}$, the netting set of trades $\hat{\tau}(\{b, c\}_i)$ is given as the netting set $\tau(\{b, b'\}_j)$, wherein each trade the side $b'$ is replaced by $c$. ♢

**Definition A.18** (compression operator)**.** The *compression operator* CMP associates to each trade relation graph $\mathrm{FS} = (B, \mathscr{L}, \tau)$ a trade relation graph $\mathrm{FS}' = (B', L', \tau')$, called the *compression of* FS, as follows:

(i) $B' := B$, i.e., the nodes in the compressed system are the same as in the original system.

[25] For example: If $\ell = \{b_1, b_2\}$, and $\tau(\ell) = \{\mathcal{T}\langle b_1, b_2\rangle\} =: \{T\}$ and $\alpha(T) = c_3$ , then $\hat{\tau}(\ell_1) = \{\mathcal{T}\langle b_1, c_3\rangle\}$ and $\hat{\tau}(\ell_2) = \{\mathcal{T}\langle c_3, b_2\rangle\}$.

(ii) $L' := L$, i.e., the links in the compressed system are the same as in the original system but with all parallel links removed.

(iii) The function $\tau'$ is defined by

$$\tau'(\{b_1, b_2\}) := \bigcup_{\{b_1,b_2\}_i \in \mathscr{L}(b_1,b_2)} \tau(\{b_1, b_2\}_i) \tag{A.19}$$

for any $\{b_1, b_2\} \in L'$, i.e., in the compressed system, the one link between $b_1$ and $b_2$ now contains the union over all netting sets, which were previously attached to the various parallel links between $b_1$ and $b_2$. ◊

**Definition A.19** ($m$-clearing operator)**.** Let $\mathrm{FS} = (B, \mathscr{L}, \tau)$ be a trade relation graph with trade data function $\tau : \mathscr{L} \to [Y]$ and $\alpha : Y \to \Gamma$. The *$m$-clearing operator* $\overline{\mathcal{MC}}^{(m)}$ with respect to $\alpha$ defined by

$$\overline{\mathcal{MC}}^{(m)} := \mathrm{CMP} \circ \widehat{\mathcal{MC}}^{(m)} \circ \mathrm{RP}_\alpha$$

associates to each such trade relation graph $\mathrm{FS} = (B, \mathscr{L}, \tau)$ a trade relation graph $\overline{\mathrm{FS}}^{(m)} = (\bar{B}, \bar{L}, \bar{\tau})$. In case $m \geqslant 2$, this graph is called the *$m$-fold multi clearing of* FS and in case $m = 1$, the operator $\overline{\mathcal{CC}} := \overline{\mathcal{MC}}^{(1)}$ is called *central clearing operator* and we denote $\overline{\mathrm{FS}} := \overline{\mathrm{FS}}^{(1)}$. ◊

**Remark A.20** (adjacency matrix)**.** The central pre-clearing and clearing of a financial system $\mathrm{FS} = (B, \mathscr{L}, \tau)$ on the graph level (i.e., ignoring the netting structure) can be expressed in terms of the graphs adjacency matrix: Recall that the adjacency matrix $A$ of a graph $(B, \mathscr{L})$ with $n = |B|$ nodes is defined as $a_{ij} = |\mathscr{L}(b_i, b_j)|$, $1 \leqslant i, j \leqslant n$, i.e., $a_{ij}$ is the number of links between node $b_i$ and node $b_j$. If $A \in \mathbb{R}^{n \times n}$ is the adjacency matrix of the bilateral system, then the matrix

$$\hat{A} := \begin{pmatrix} & & & \deg(b_1) \\ & \mathbf{0_n} & & \cdots \\ & & & \deg(b_n) \\ \deg(b_1) & \ldots & \deg(b_n) & 0 \end{pmatrix} \in \mathbb{R}^{(n+1)\times(n+1)}$$

is the adjacency matrix of the central pre-clearing of FS and

$$\bar{A} := \begin{pmatrix} & & & 1 \\ & \mathbf{0_n} & & \cdots \\ & & & 1 \\ 1 & \cdots & 1 & 0 \end{pmatrix} \in \mathbb{R}^{(n+1)\times(n+1)} \tag{A.20}$$

is the adjacency matrix of the clearing of FS. In both cases, we assume that the CCP is added as node $n+1$. An analogous representation is possible for $m$-fold multi-(pre-)-clearing; in that case, $m$ rows/columns must be added.

Expressing a graph as an adjacency matrix is a common tool in graph theory. The eigenvalues of the adjacency matrix are called the *spectrum* of a graph. Because of its particularly simple structure, one can compute the eigenvalues of Eq. (A.20) analytically as $\{0, \pm\sqrt{n-1}\}$, see Theorem A.6. More on spectral graph theory can be found in textbooks such as [10]. ◊

**Theorem A.21** (risk graph and central (pre-)clearing)**.** Let $\mathrm{FS} = (B, \mathscr{L}, \tau)$ be a bilateral trade relation graph with trade data function $\tau : \mathscr{L} \to [Y]$ and assume that FS is $\alpha$-partitioned with respect to a function $\alpha : Y \to \Gamma = \{\gamma_1, \ldots, \gamma_m\}$. Let $w$ be a bilateral exposure metric (in the sense of Definition A.12). Then the risk graph $\mathrm{RG} = (B, \mathscr{A}, w)$ of FS, the risk graph $\widehat{\mathrm{RG}}^{(m)} = (\hat{B}, \hat{\mathscr{A}}, \hat{w})$ of its $m$-pre-clearing $\widehat{\mathcal{MC}}^{(m)}(\mathrm{FS})$ and the risk graph $\overline{\mathrm{RG}}^{(m)} = (\bar{B}, \bar{A}, \bar{w})$ of its $m$-clearing $\overline{\mathcal{MC}}^{(m)}(\mathrm{FS})$ are related as follows:

(i) Nodes: All three risk graphs contain the nodes $B$, but $\widehat{\mathrm{RG}}^{(m)}$ and $\overline{\mathrm{RG}}^{(m)}$ contain the CCP nodes in addition, i.e.

$$\bar{B} = \hat{B} = B \dot{\sqcup} \{c_1, \ldots, c_m\}.$$

(ii) Links (pre-clearing): For any link $\ell = \{b_1, b_2\}_i \in \mathscr{L}$ in the bilateral system FS, there are precisely four arrows $(b_1, c_\mu)_{\hat{j}}$, $(c_\mu, b_1)_{\hat{j}}$, $(b_2, c_\mu)_{\hat{j}}$, $(c_\mu, b_2)_{\hat{j}}$ in $\hat{\mathscr{A}}$.

Links (clearing): For any link $\ell = \{b_1, b_2\}_i \in \mathscr{L}$ pre-cleared through $c_\mu$, there are precisely two arrows $(b_j, c_\mu), (c_\mu, b_j) \in \bar{A}$, $j = 1, 2$.

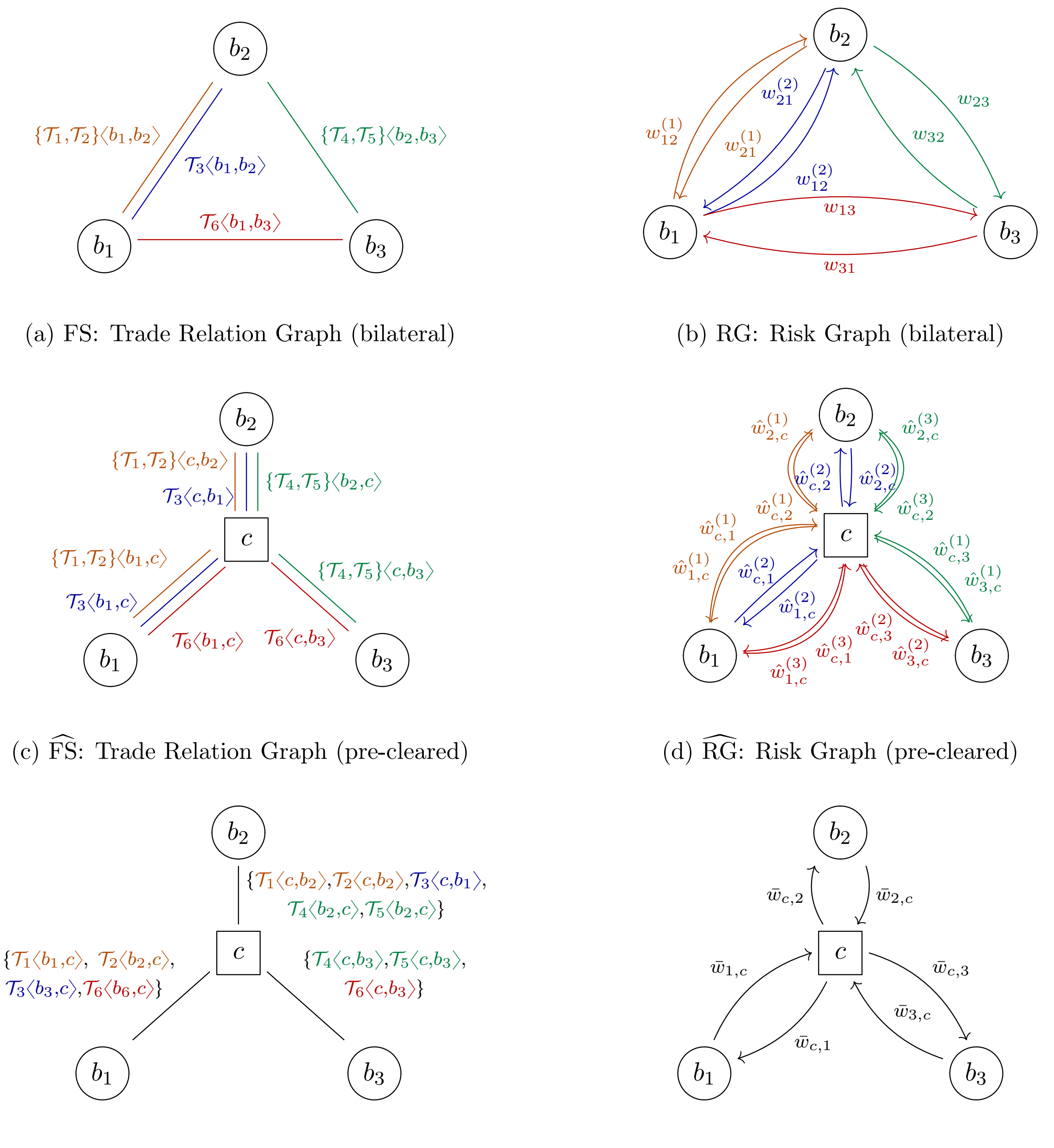


(a) FS: Trade Relation Graph (bilateral)

(b) RG: Risk Graph (bilateral)

(c) $\widehat{\text{FS}}$: Trade Relation Graph (pre-cleared)

(d) $\widehat{\text{RG}}$: Risk Graph (pre-cleared)

(e) $\overline{\text{FS}}$: Trade Relation Graph (cleared)

(f) $\overline{\text{RG}}$: Risk Graph (cleared)

Figure 18: Pre-clearing and clearing a bilateral system (colors indicate netting sets)

(iii) Degrees: The node degrees[26] satisfy for any $b \in B$,

$$\widehat{\deg}(b) = \deg(b) = \deg^{\pm}(b) = \widehat{\deg}^{\pm}(b) \tag{A.21}$$

$$\overline{\deg}(b) = \overline{\deg}^{\pm}(b) = |\Gamma(b)|, \tag{A.22}$$

[26]For any bank $b$, we denote by $\deg(b)$, the degree of $b$ in FS, by $\widehat{\deg}(b)$, the degree of $b$ in $\widehat{\text{FS}}^{(m)}$ and by $\overline{\deg}(b)$, the degree of $b$ in $\overline{\text{FS}}^{(m)}$. Analogously, we denote by $\deg^{\pm}(b)$, $\widehat{\deg}^{\pm}(b)$ respectively $\overline{\deg}^{\pm}(b)$, the in/out-degree of $b$ in RG, $\widehat{\text{RG}}^{(m)}$, respectively $\overline{\text{RG}}^{(m)}$.

where $|\Gamma(b)|$ is the number of trade types occurring in the links at $b$. The CCPs satisfy

$$\sum_{k=1}^{m} \widehat{\deg}^{\pm}(c_k) = \sum_{k=1}^{m} \widehat{\deg}(c_k) = |\hat{\mathscr{L}}| = 2|\mathscr{L}| \tag{A.23}$$

$$\sum_{k=1}^{m} \overline{\deg}^{\pm}(c_k) = \sum_{k=1}^{m} \overline{\deg}(c_k) = |\bar{L}| = \sum_{b\in B} |\Gamma(b)|. \tag{A.24}$$

(iv) Risks (links): For any link $\ell = \{b_1, b_2\}_i \in \mathscr{L}$ such that $\alpha(\ell) = c_k$, the associated risk weights for the bilateral system in RG and the $m$-pre-cleared system in $\widehat{\mathrm{RG}}^{(m)}$ are related by

$$w_{1,2}^{(i)} = \hat{w}_{1,c_k}^{(i)} = \hat{w}_{c_k,2}^{(i)} \tag{A.25}$$

$$w_{2,1}^{(i)} = \hat{w}_{2,c_k}^{(i)} = \hat{w}_{c_k,1}^{(i)}, \tag{A.26}$$

whereas the risk weights in the $m$-cleared system $\overline{\mathrm{RG}}^{(m)}$ satisfy

$$\bar{w}_{1,c_k} \leqslant \sum_i w_{1,c_k}^{(i)} \tag{A.27}$$

$$\bar{w}_{c_k,1} \leqslant \sum_i w_{c_k,1}^{(i)}. \tag{A.28}$$

(v) Risks (nodes): The aggregations of the risk weights from the links to the nodes satisfy

$$\forall b \in B : \bar{w}^{\pm}(b) \leqslant \hat{w}^{\pm}(b) = w^{\pm}(b), \tag{A.29}$$

$$\forall 1 \leqslant k \leqslant m : \bar{w}^{\pm}(c_k) \leqslant \hat{w}^{\pm}(c_k). \tag{A.30}$$

(vi) Risks (banks vs. CCPs): The total risk induced by all banks is the risk received by the CCP and vice versa:

$$\sum_{k=1}^{m} \hat{w}^{\pm}(c_k) = \sum_{b\in B} \hat{w}^{\mp}(b) = w(\mathrm{RG}), \tag{A.31}$$

$$\sum_{k=1}^{m} \bar{w}^{\pm}(c_k) = \sum_{b\in B} \bar{w}^{\mp}(b). \tag{A.32}$$

(vii) Risks (CCPs vs totals): The total amount of risk in the system can be entirely expressed with

respect to the CCPs:

$$\hat{w}(\widehat{\mathrm{RG}}^{(m)}) = \sum_{k=1}^{m} \hat{w}^{+}(c_k) + \hat{w}^{-}(c_k), \tag{A.33}$$

$$\bar{w}(\overline{\mathrm{RG}}^{(m)}) = \sum_{k=1}^{m} \bar{w}^{+}(c_k) + \bar{w}^{-}(c_k). \tag{A.34}$$

(viii) Risks (system, totals): The aggregations of risk to the system totals satisfy the following:

$$0 \leqslant \bar{w}(\overline{\mathrm{RG}}^{(m)}) \leqslant \hat{w}(\widehat{\mathrm{RG}}^{(m)}) = 2w(\mathrm{RG}). \tag{A.35}$$

(ix) Risks (system, relative): The relative risk metrics on the nodes satisfy

$$\hat{\rho}^{\pm}(b) = \tfrac{1}{2}\rho^{\pm}(b), \tag{A.36}$$

$$\hat{\rho}^{\pm}(\{c_1, \dots, c_m\}) = \tfrac{1}{2}. \tag{A.37}$$

(x) Risks (system, max): For $m = 1$, the remaining systemic risk metrics satisfy

$$\max_{b' \in B \cup \{c\}} \hat{w}^{\pm}(b') = \hat{w}^{\pm}(c) = w(\mathrm{RG}), \tag{A.38}$$

$$\max_{b' \in B \cup \{c\}} \hat{\rho}^{\pm}(b') = \hat{\rho}^{\pm}(c) = \tfrac{1}{2}. \tag{A.39}$$

◊

**Proof.** Claims (i) and (ii) about the nodes and the links follow directly from Definitions A.16 and A.19 of $m$-(pre)-clearing and of the risk graph operator, recall Definition A.8.

Claim (iii) can be seen as follows: By Definition A.16 of the $m$-pre-clearing, any bank $b \in B$ has the same number of links as in the bilateral system (but with the CCPs instead of the counterparties), thus $\widehat{\deg}(b) = \deg(b)$, which together with Eq. (A.12) yields Eq. (A.21). In the $m$-cleared system, any bank $b$ has precisely one link to each class of trades that occurred in all its netting sets, which together with Eq. (A.12) proves Eq. (A.22). In the $m$-pre-clearing, any bank can only have links with the CCPs, i.e., every link is connected to precisely one CCP, thus $\sum_{k=1}^{m} \widehat{\deg}(c_k) = |\hat{\mathscr{L}}|$. By definition of $m$-pre-clearing, every trade link in the bilateral system is broken into precisely two trade links in the $m$-pre-clearing, thus $|\hat{\mathscr{L}}| = 2|\mathscr{L}|$, which yields Eq. (A.23). In the $m$-clearing, every link is connected to precisely one CCP, thus $\sum_{k=1}^{m} \overline{\deg}(c_k) = |\bar{L}|$, and every bank $b$ is connected to precisely $|\Gamma(b)|$ CCPs, thus $|\bar{L}| = \sum_{b \in B} |\Gamma(b)|$.

Claim (iv) follows from Definition A.12 of a bilateral exposure metric.

Claim (v) follows from claim (iv).

Claim (vi) follows from the fact that in the $m$-(pre-)cleared system, all the banks are only connected to the CCPs (but not amongst each other), and all the CCPs are only connected to the banks (but not with each other).

Claim (vii) follows from claim (vi).

The key claim (viii) now follows from (v):

$$\begin{aligned}
\bar{w}(\overline{\mathrm{RG}}^{(m)}) &= \sum_{k=1}^{m} \bar{w}^{\pm}(c_k) + \sum_{b\in B} \bar{w}^{\pm}(b) \\
&\leqslant \underbrace{\sum_{k=1}^{m} \hat{w}^{\pm}(c_k) + \sum_{b\in B} \hat{w}^{\pm}(b)}_{=\hat{w}(\widehat{\mathrm{RG}}^{(m)})} \\
&= \sum_{b\in B} \hat{w}^{\mp}(b) + \sum_{b\in B} w^{\pm}(b) \\
&= 2w(\mathrm{RG}).
\end{aligned}$$

Claim (ix) follows from Eq. (A.29) and Eq. (A.35) via

$$\hat{\rho}^{\pm}(b) = \frac{\hat{w}^{\pm}(b)}{\hat{w}(\widehat{\mathrm{RG}}^{(m)})} = \frac{w^{\pm}(b)}{2w(\mathrm{RG})} = \frac{1}{2}\rho^{\pm}(b)$$

and from Eq. (A.31) via

$$\hat{\rho}^{\pm}(b) = \frac{\hat{w}^{\pm}(b)}{\hat{w}(\widehat{\mathrm{RG}}^{(m)})} = \frac{w^{\pm}(b)}{2w(\mathrm{RG})} = \frac{1}{2}\rho^{\pm}(b).$$

Claim (x) follow from Eq. (A.37). □

**Definition A.22** ($w$-reducing)**.** Using the notation of Theorem 5.1, we say that the central clearing operator is $w$-reducing on FS if

$$\Delta_w(\mathrm{FS}) := w(\mathrm{RG}) - \bar{w}(\overline{\mathrm{RG}}) > 0. \tag{A.40}$$

The quantity $\Delta_w(\mathrm{FS})$ is called the *netting benefit in* FS. ◊

**Definition A.23** (netting benefit)**.** Using the notation of Theorem 5.1 (for $m = 1$), for any bank

$b \in B$, we call

$$\Delta_w^{\pm}(b) := w^{\pm}(b) - \bar{w}^{\pm}(b), \tag{A.41}$$

$$r_w^{\pm}(b) := \begin{cases} \frac{\bar{w}^{\pm}(b)}{w^{\pm}(b)}, & w^{\pm}(b) \neq 0, \\ 1, & w^{\pm}(b) = 0. \end{cases} \tag{A.42}$$

the *absolute* respectively *relative netting benefit (of central clearing) at b*. We also define the absolute and relative netting benefit of the whole system by

$$\Delta_w^{\pm}(\mathrm{RG}) = w(\mathrm{RG}) - \bar{w}(\overline{\mathrm{RG}}), \tag{A.43}$$

respectively

$$r_w(\mathrm{RG}) := \begin{cases} \frac{\bar{w}(\overline{\mathrm{RG}})}{w(\mathrm{RG})}, & w(\mathrm{RG}) \neq 0, \\ 1, & w(\mathrm{RG}) = 0 \end{cases} < 1. \tag{A.44}$$

◊

**Theorem A.24.** Using the notation of Theorem 5.1 (for $m = 1$), any of the following are equivalent to $w$-reducing:

(i) $\Delta_w(\mathrm{RG}) > 0$.

(ii) $r_w(\mathrm{RG}) < 1$.

(iii) $\sum_{b \in B} \Delta_w^{\pm}(b) > \bar{w}^{\pm}(c)$.

(iv) $\sum_{b \in B} r_w^{\pm}(b)\rho^{\pm}(b) + r_w^{\mp}(b)\rho^{\mp}(b) < 1$ ◊

**Proof.** The equivalence between (i) and (ii) is obvious. The equivalence between (i) and (iii) follows from

$$\begin{aligned} w(\mathrm{RG}) - \bar{w}(\overline{\mathrm{RG}}) &= \sum_{b \in B} w^{\pm}(b) - \bar{w}^{\pm}(b) - \bar{w}^{\pm}(c) \\ &= \sum_{b \in B} \Delta_w^{\pm}(b) - \bar{w}^{\pm}(c). \end{aligned}$$

The equivalence between (ii) and (iv) follows from Eq. (A.32) via:

$$\begin{aligned} r_w(\mathrm{RG}) &= \frac{\sum_{b\in B} \bar{w}^{\pm}(b) + \bar{w}^{\pm}(c)}{\sum_{b\in b} w^{\pm}(b)} \\ &= \sum_{b\in B} \frac{\bar{w}^{\pm}(b)}{w^{\pm}(b)} \frac{w^{\pm}(b)}{\sum_{b\in b} w^{\pm}(b)} + \frac{\bar{w}^{\pm}(c)}{\sum_{b\in b} w^{\pm}(b)} \\ &= \sum_{b\in B} r_w^{\pm}(b)\rho^{\pm}(b) + \sum_{b\in B} \frac{\bar{w}^{\mp}(b)}{\sum_{b\in b} w^{\mp}(b)} \\ &= \sum_{b\in B} r_w^{\pm}(b)\rho^{\pm}(b) + r_w^{\mp}(b)\rho^{\mp}(b). \end{aligned}$$

□

**Theorem A.25** (role of the CCP)**.** Using the notation of Theorem 5.1, the following hold:

(i) The CCP knows the netting benefits of every bank $b \in B$:

$$\Delta_w^{+}(b) = \sum_{\{b,c\}_i \in \hat{\mathscr{L}}(b,c)} \hat{w}_{bc}^{(i)} - \bar{w}_{bc}, \tag{A.45}$$

$$\Delta_w^{-}(b) = \sum_{\{b,c\}_i \in \hat{\mathscr{L}}(b,c)} \hat{w}_{cb}^{(i)} - \bar{w}_{cb}. \tag{A.46}$$

(ii) The netting benefit of the system is entirely determined by the netting benefits of the CCP:

$$\Delta_w(\mathrm{FS}) = \Delta_w^{\mp}(c) - \bar{w}^{\pm}(c). \tag{A.47}$$

◊

**Proof.** Claim (i) follows directly from the definitions. To see (ii), we compute

$$\begin{aligned} \Delta_w(\mathrm{FS}) &= \sum_{b\in B} \hat{w}^{\pm}(b) - \bar{w}^{\pm}(b) - \bar{w}^{\pm}(c) \\ &\overset{\text{(A.31),(A.32)}}{=} \hat{w}^{\mp}(c) - \bar{w}^{\mp}(c) - \bar{w}^{\pm}(c) \\ &= \Delta_w^{\mp}(c) - \bar{w}^{\pm}(c). \end{aligned}$$

□

**Corollary A.26** (netting benefit symmetries)**.** Under the hypothesis of Theorem 5.1 (for $m = 1$), it holds that

$$\sum_{b\in B} \Delta_w^{\pm}(b) = \Delta_w^{\pm}(c), \tag{A.48}$$

if the bilateral exposure metric $w$ is chosen as $w \in \{V^{\pm}(t,\omega), \mathrm{EPE}(t), \mathrm{ENE}(t), \mathrm{EEPE}\}$, c.f. Re-

mark A.13. In particular, $\bar{\rho}^{\pm}(c) = \frac{1}{2}$. ◊

**Proof.**

Step 1: For any $x \in \mathbb{R}$, we define $x^+ := \max(x, 0)$ and $x^- := -\min(x, 0) = (-x)^+$. For a vector $v = (v_1, \ldots, v_n) \in \mathbb{R}^n$, we define its *netting benefit* by

$$\mathcal{N}^{\pm}(v) := \sum_{i=1}^{n} v_i^{\pm} - \Big(\sum_{i=1}^{n} v_i\Big)^{\pm}.$$

The proof rests on the key fact that

$$\forall n \geqslant 2 : \forall v \in \mathbb{R}^n : \mathcal{N}^{\pm}(v) = \mathcal{N}^{\pm}(-v). \tag{A.49}$$

This follows from the calculation

$$\begin{aligned}
&\mathcal{N}^{+}(v) - \mathcal{N}^{+}(-v) \\
&= \sum_{i=1}^{n} (v_i^{+} - (-v_i)^{+}) - \Big(\Big(\sum_{i=1}^{n} v_i\Big)^{+} - \Big(-\sum_{i=1}^{n} v_i\Big)^{+}\Big) \\
&= \sum_{i=1}^{n} v_i - \sum_{i=1}^{n} v_i = 0,
\end{aligned}$$

since $x = x^+ - (-x)^+$ for any $x \in \mathbb{R}$. Applying this to $-x$ instead yields the result for $\mathcal{N}^-$.

Step 2: Now, let $b$ be a bank and $c$ be the CCP and assume that in the pre-cleared system $\widehat{\mathrm{FS}}$, they have $k$ netting sets and their value processes (from the perspective of $b$) are labeled $V = (V_1, \ldots, V_k)$. Then at any time $t$ and in any path $\omega$, the netting benefit of $b$ measured in the metric $w = V(t, \omega)^{\pm}$ is given by $\mathcal{N}^{\pm}(V(t, \omega))$. By Eq. (A.49), the netting benefit of $b$ is, therefore, the same as the netting benefit of $c$ on these $k$ netting sets as their value process from the perspective of $c$ is given by $-V$. By applying this to all banks $b \in B$, we obtain Eq. (A.48). This implies

$$\begin{aligned}
\bar{w}(\overline{\mathrm{FS}}) &= \sum_{b \in B} \bar{w}^{\pm}(b) + \bar{w}^{\pm}(c) \\
&= \sum_{b \in B} \hat{w}^{\pm}(b) - \Delta_w^{\pm}(b) + \hat{w}^{\pm}(c) - \Delta_w^{\pm}(c) \\
&= \sum_{b \in B} \hat{w}^{\pm}(b) + \hat{w}^{\pm}(c) - 2\Delta_w^{\pm}(c) \\
&= \hat{w}(\widehat{\mathrm{FS}}) - 2\Delta_w^{\pm}(c).
\end{aligned}$$

In the pre-cleared system, we know that $\hat{\rho}^{\pm}(c) = \frac{1}{2}$ by Eq. (A.37). This implies

$$\begin{aligned}
&\tfrac{1}{2} = \hat{\rho}^{\pm}(c) = \tfrac{\hat{w}^{\pm}(c)}{\hat{w}(\widehat{\mathrm{FS}})} \\
\Longrightarrow & 2\hat{w}^{\pm}(c) = \hat{w}(\widehat{\mathrm{FS}}) \\
\Longrightarrow & 2(\hat{w}^{\pm}(c) - \Delta_w^{\pm}(c)) = \hat{w}(\widehat{\mathrm{FS}}) - 2\Delta_w^{\pm}(c) \\
\Longrightarrow & 2\bar{w}^{\pm}(c) = \bar{w}(\overline{\mathrm{FS}}) \\
\Longrightarrow & \bar{\rho}^{\pm}(c) = \tfrac{1}{2}.
\end{aligned}$$

Step 3: We have proven the claim for the metric $w = V^{\pm}(t, \omega)$ for any $t > 0$ and $\omega$. Therefore, the claim also holds for its expectation, which proves the claim for $w = \mathrm{EPE}(t)$ and $w = \mathrm{ENE}(t)$. This, in turn, proves the claim for EEPE. □

## A.5 Additional Case Studies

### A.5.1 A star is cleared

Central clearing introduces a big player into the system and transforms the resulting trade relation graph into a star. But what if the bilateral system is already star-shaped? We now consider a financial system with one large bank $b_0$ having one trade each with four other smaller banks $b_1, \dots, b_4$, see Fig. 19a. This trade relation graph is a *star*. If we compute the central clearing, we arrive at Fig. 19c. All the smaller banks $b_1, \dots, b_4$ have the same trades as before. However, $b_0$ now has one big netting set with the clearing house. We conclude that

$$\bar{w}^{\pm}(b_i) = w^{\pm}(b_i), \qquad i = 1, 2, 3, 4, \tag{A.50}$$

i.e., the small banks have the same risks as before, but $\bar{w}^{\pm}(b_0) \leqslant \hat{w}^{\pm}(b_0)$, i.e., the large bank may realize netting benefits. We conclude from Fig. 19b that the bilateral system satisfies

$$w(\mathrm{RG}) = w^{+}(b_0) + \sum_{i=1}^{4} w^{+}(b_i) = \sum_{i=1}^{4} w^{-}(b_i) + w^{+}(b_i).$$

In the cleared system, see Fig. 19d, we have

$$\bar{w}^{+}(c) = \bar{w}_{c0} + \sum_{i=1}^{4} \bar{w}_{ci} = \bar{w}_{c0} + \sum_{i=1}^{4} w^{-}(b_i),$$

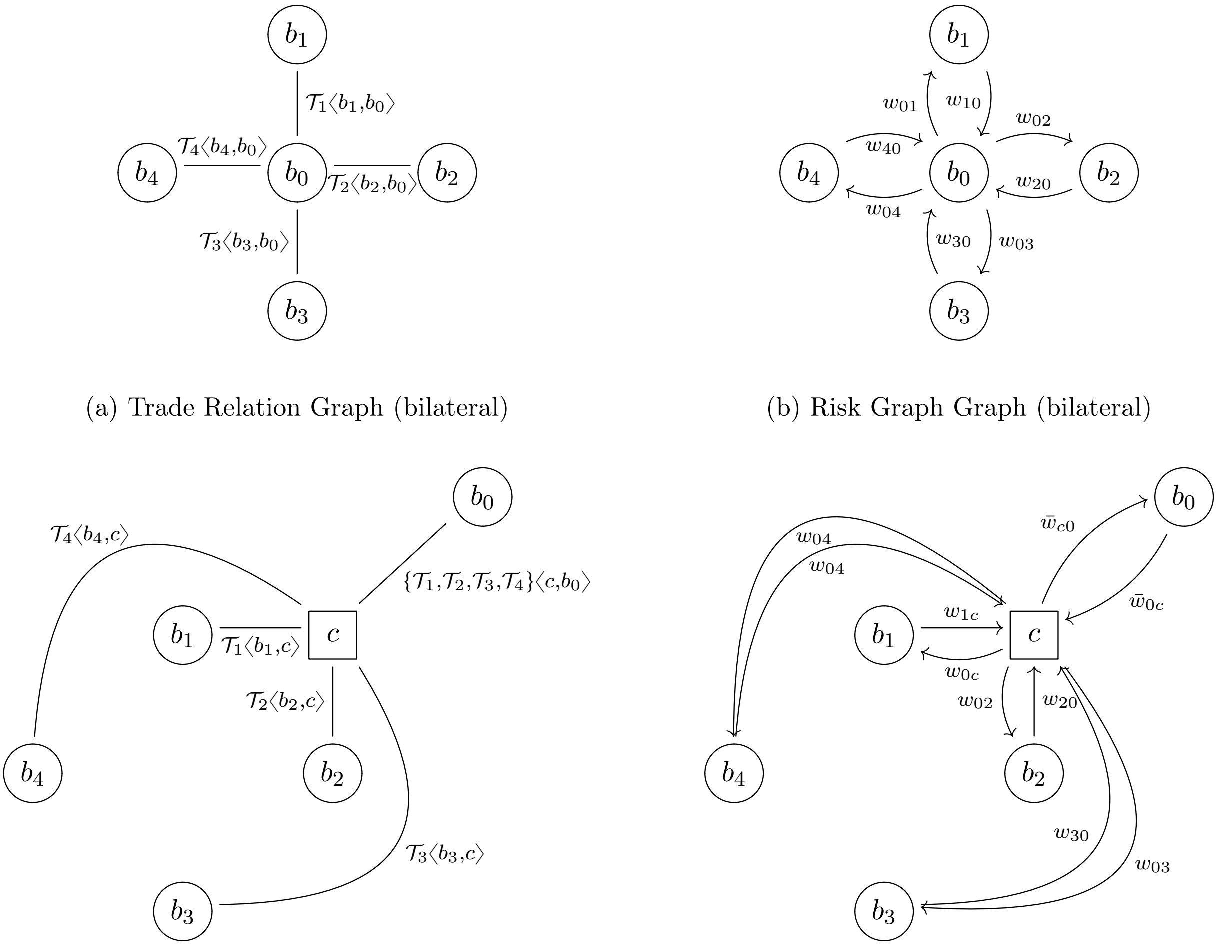


(a) Trade Relation Graph (bilateral)

(b) Risk Graph Graph (bilateral)

(c) Trade Relation Graph (cleared)

(d) Risk Graph (cleared)

Figure 19: A star is cleared

thus,

$$
\begin{aligned}
\bar{w}(\overline{\mathrm{RG}}) &= \bar{w}^+(b_0) + \bar{w}^+(c) + \sum_{i=1}^{4} \bar{w}^+(b_i) \\
&= \bar{w}_{0c} + \bar{w}_{c0} + \sum_{i=1}^{4} w^+(b_i) + w^-(b_i) \\
&= w(\mathrm{RG}) + \bar{w}_{0c} + \bar{w}_{c0} \geqslant w(\mathrm{RG})
\end{aligned}
$$

with equality if and only if $w_{0c} + w_{c0} = 0$, i.e. if the netting set $\{\mathcal{T}_1, \mathcal{T}_2, \mathcal{T}_3, \mathcal{T}_4\}$ cancels out. This might be considered a realistic scenario if $b_0$ has deliberately constructed these trades in pairs of hedges. In all other cases, $\bar{w}(\overline{\mathrm{RG}}) > w(\mathrm{RG})$, i.e., the bilateral system is safer than the cleared system.

We conclude that clearing a star-shaped system is never beneficial. A star-shaped system is a simplified yet prototypical example of the old bilateral world in which several smaller players trade through a big central intermediary. The economic consequence is that applying central clearing to

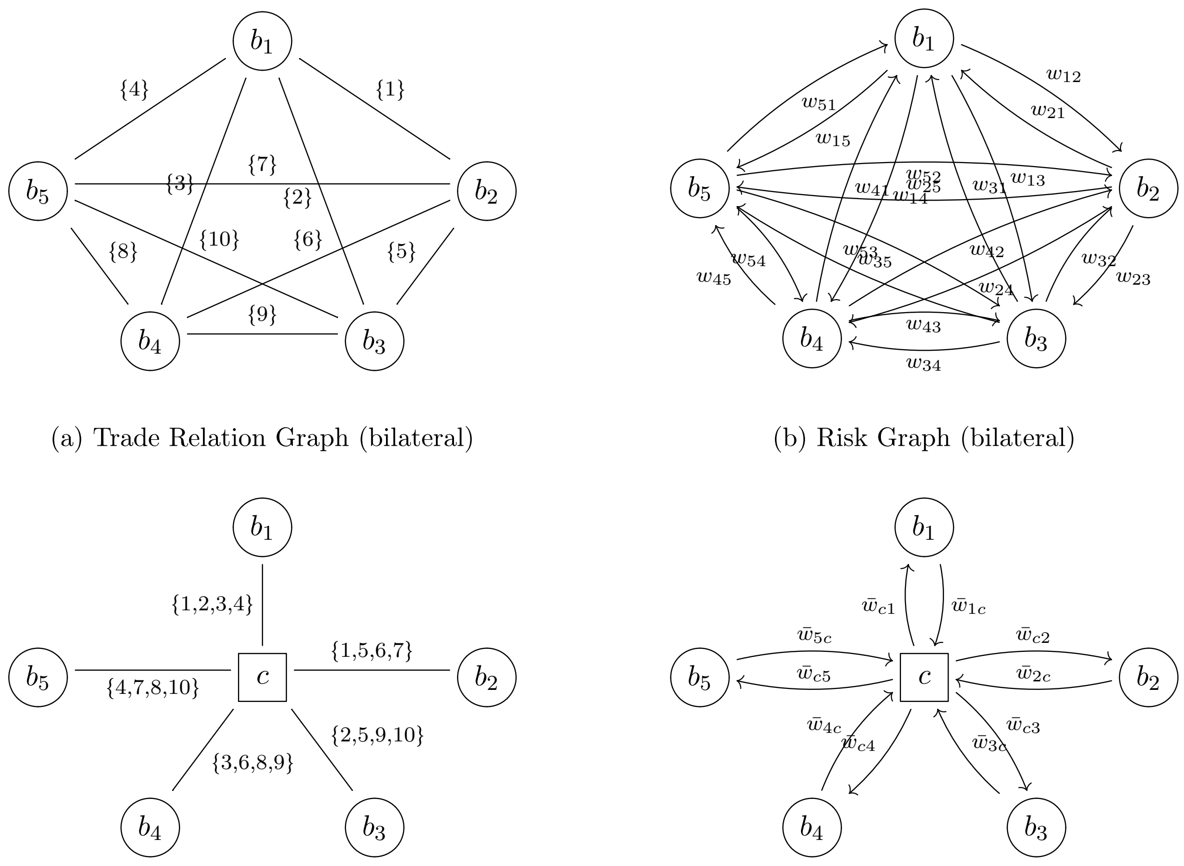


(a) Trade Relation Graph (bilateral) (b) Risk Graph (bilateral)

(c) Trade Relation Graph (cleared) (d) Risk Graph (cleared)

Figure 20: Clearing a complete graph

an already "centralized" situation is not very beneficial. Given that a cleared system is always star-shaped, this hints that applying a central clearing operator twice, i.e., clearing a cleared system, is not beneficial either.

This example can be seen as an economic companion to Fig. 6a, a prototypical example of several equal counterparties trading bilaterally with each other without a central intermediary. In that situation, the central clearing was hugely beneficial.

Mathematically, this example is also interesting because a star has minimal links required to be connected, i.e., removing any link from the trade relation graph Fig. 19a would make the graph disconnected.

### A.5.2 Complete Graphs

In this section, we study the opposite, i.e., a trade relation graph in which all nodes have as many links as possible but no parallel links. Such a graph is called *complete*, see Fig. 20. In such a bilateral system, each of the 5 banks has 4 netting sets, and thus we expect that in the cleared system, it can realize many netting benefits. However, mathematically, we only obtain $\bar{w}^{\pm}(b_i) \leqslant w^{\pm}(b_i)$, $i = 1, \dots, 5$,

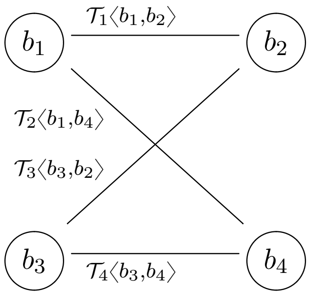


(a) Trade Relation Graph (bilateral)

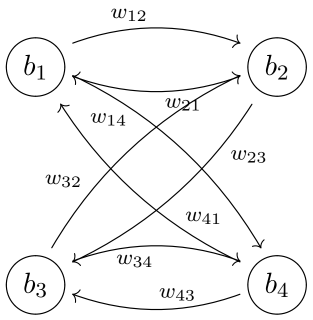


(b) Risk Graph (bilateral)

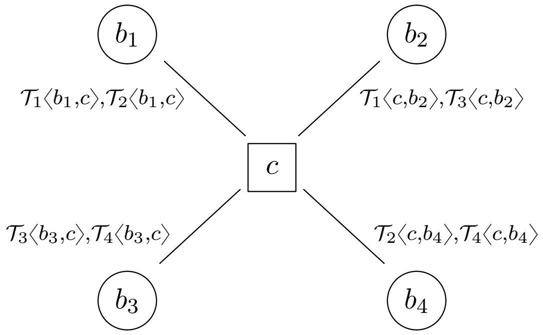


(c) Trade Relation Graph (cleared)

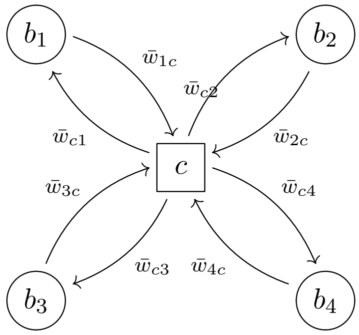


(d) Risk Graph (cleared)

Figure 21: Bipartite

i.e., for any bank $b_i$ the total amount of risk might go down, but it might also be the same depending on whether or not the trades net out a lot or not at all. In particular, it cannot be concluded a priori that the sum of all netting benefits is enough to conclude that $\bar{w}(\overline{\text{RG}}) < w(\text{RG})$. However, we would expect this to hold if the system contains many hedged trades.

Among the connected graphs, there are two extremes: A complete graph has as many edges as possible (but none parallel) because every vertex is connected with every other vertex. A *tree* has as few edges as needed to be connected (a star is a special case of a tree). For the star, we can prove that $\bar{w}(\overline{\text{RG}}) \geqslant w(\text{RG})$ and for the complete graph, we cannot prove a priori, i.e., without detailed knowledge of the netting sets, that central clearing makes this system safer or not. In reality, the financial system comprises a few big players who trade as equals with each other, and each of those players also trades with many small players. Therefore, in reality, we expect effects from parts of the financial system that are complete or have a high interconnectedness overlay with effects from nearly star-shaped parts. It is impossible to answer a priori whether central clearing would make such a system safer. This highlights the need for a detailed quantitative study of these effects via numerical simulation, see Section 7.

### A.5.3 Bipartite Graph

A generalization of the example with the two banks in Section 6.1 is provided by a financial system that has a *bipartite* trade relation graph $\mathrm{FS} = (B, \mathscr{L}, \tau)$. Recall that a graph is *bipartite*, if its nodes $B$ can be divided into two disjoint groups $B = B_1 \dot{\cup} B_2$ and any link $\ell \in \mathscr{L}$ always connects a node from $B_1$ with a node from $B_2$, but never any two nodes within $B_1$ or within $B_2$. An example of a bipartite system with $n = 4$ nodes is shown in Fig. 21a, where the banks give the two groups with the odd labels $b_1, b_3$ and the even labels $b_2, b_4$. That system's risk graph and clearing are shown in Fig. 21.

A bipartite graph topology has the property that the impact of the clearing operator can be both maximally beneficial or adverse, depending on how the trades are distributed. On the one extreme, if all the trades in the system are distributed such that all banks in the one group have the deals that are in the money, the other group has the side that is out of the money. It is difficult for the central clearing operator to realize the netting benefits. On the other hand, if both groups are well balanced, many trades are hedged, and thus clearing realizes many benefits.

To illustrate this, let's consider two extremes: If all four trades $\mathcal{T}_1, \mathcal{T}_2, \mathcal{T}_3, \mathcal{T}_4$ are equal to $\mathcal{T}$, i.e., $b_1$ has $\mathcal{T}\langle b_1, b_2\rangle$ and $\mathcal{T}\langle b_1, b_4\rangle$ and $b_3$ also has $\mathcal{T}\langle b_3, b_2\rangle$ and $\mathcal{T}\langle b_3, b_4\rangle$, then absolutely no netting benefits can be realized by any of the banks or the CCP. Thus, in this case $\bar{w}(\overline{\mathrm{RG}}) = \hat{w}(\widehat{\mathrm{RG}}) = 2w(\mathrm{RG})$. On the other extreme, if $b_1$ has a deal $\mathcal{T}$ with $b_2$, i.e., $\mathcal{T}\langle b_1, b_2\rangle$ and its perfect hedge $\overline{\mathcal{T}}$ with $b_4$, i.e., $\overline{\mathcal{T}}\langle b_2, b_4\rangle$, and $b_3$ has $\overline{\mathcal{T}}\langle b_3, b_2\rangle$ and $\mathcal{T}\langle b_3, b_4\rangle$, then all banks in the system are perfectly hedged, thus $\bar{w}(\overline{\mathrm{RG}}) = 0$. Notice that a pre-cleared or cleared system is also always an example of a bipartite graph, where the banks and the CCPs constitute the two groups of nodes.
